# Maternal effects drive intestinal development beginning in the embryonic period on the basis of maternal immune and microbial transfer in chickens

**DOI:** 10.1186/s40168-023-01490-5

**Published:** 2023-03-03

**Authors:** Haizhou Gong, Taiping Wang, Min Wu, Qianran Chu, Hainan Lan, Wuying Lang, Lingyu Zhu, Yang Song, Yujie Zhou, Qiongyi Wen, Jing Yu, Baolin Wang, Xin Zheng

**Affiliations:** 1 College of Animal Science and Technology, Jilin Agricultural University, Changchun, 130118 China; 2 Key Laboratory of Animal Production, Product Quality and Security (Jilin Agricultural University), Ministry of Education, Changchun, 130118 China

**Keywords:** Maternal effects, Maternal microbiota, Reproductive system microbiota, Embryonic gut microbiota, Maternal immune transfer, Intestinal development, Immune development, Embryonic development, Poultry

## Abstract

**Background:**

Nutrition drives immunity and health in animals, and maternal immunity benefits offspring. In our previous study, a nutritional intervention strategy was found to promote the immunity of hens, which subsequently improved immunity and growth in offspring chicks. Maternal effects clearly exist, but how are mothers’ immune advantages transferred to their offspring, and how do they benefit them?

**Results:**

Here, we traced the beneficial effects back to the process of egg formation in the reproductive system, and we focused on the embryonic intestinal transcriptome and development, as well as on maternal microbial transfer in offspring. We found that maternal nutritional intervention benefits maternal immunity, egg hatching, and offspring growth. The results of protein and gene quantitative assays showed that the transfer of immune factors into egg whites and yolks depends on maternal levels. Histological observations indicated that the promotion of offspring intestinal development begins in the embryonic period. Microbiota analyses suggested that maternal microbes transfer to the embryonic gut from the magnum to the egg white. Transcriptome analyses revealed that offspring embryonic intestinal transcriptome shifts are related to development and immunity. Moreover, correlation analyses showed that the embryonic gut microbiota is correlated with the intestinal transcriptome and development.

**Conclusions:**

This study suggests that maternal immunity positively influences offspring intestinal immunity establishment and intestinal development beginning in the embryonic period. Adaptive maternal effects might be accomplished via the transfer of relatively large amounts of maternal immune factors and by shaping of the reproductive system microbiota by strong maternal immunity. Moreover, reproductive system microbes may be useful resources for the promotion of animal health.

Video Abstract

**Graphical Abstract:**

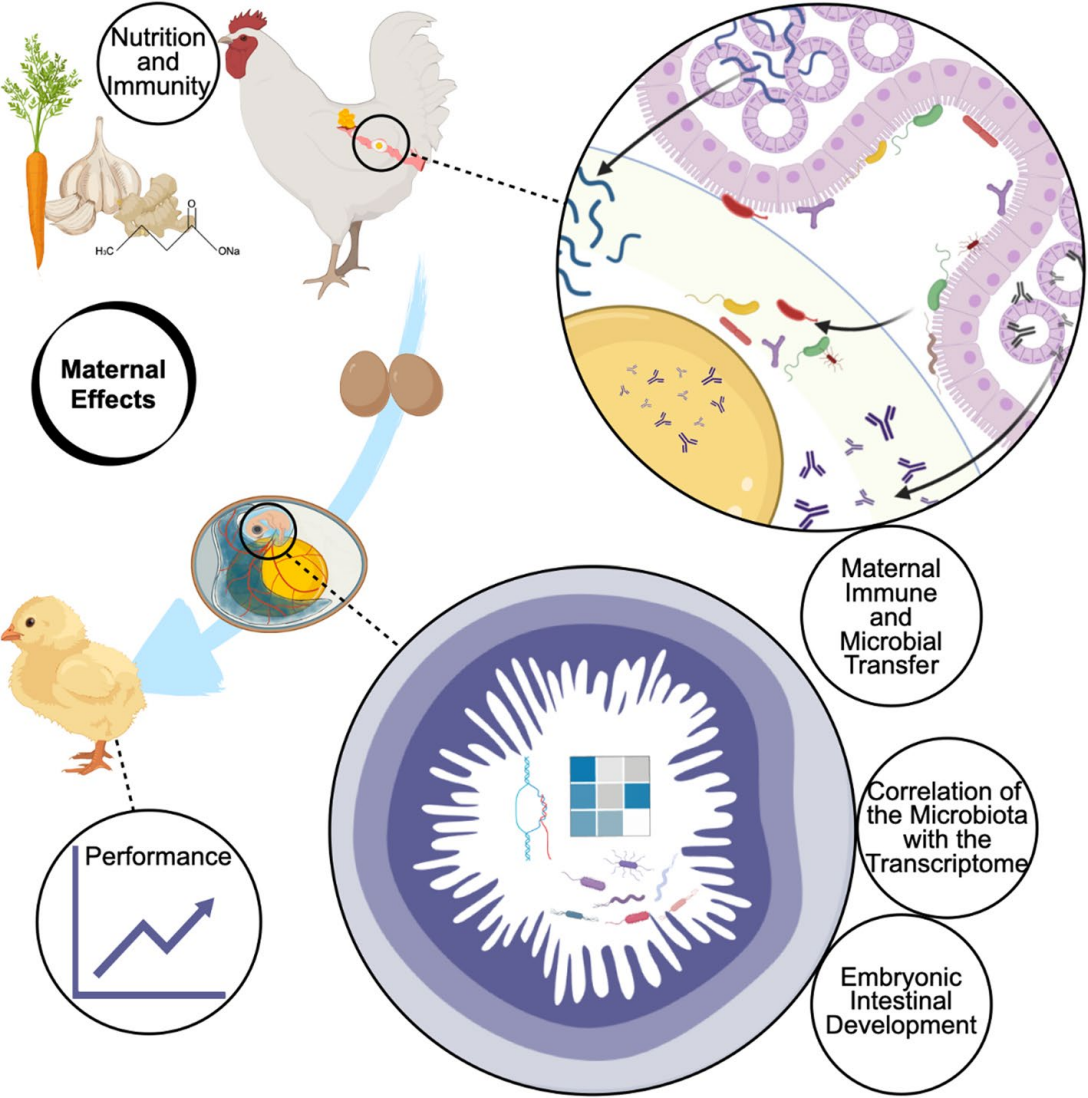

**Supplementary Information:**

The online version contains supplementary material available at 10.1186/s40168-023-01490-5.

## Background

Nutrition shapes the animal immune system and health status at many levels and involves nutrient metabolism between the host and microbiota, as well as interactions between the microbiota and the host immune system [1, 2]. To date, there is limited information on the mechanisms and pathways involved in maternal effects [3]. It is accepted that the maternal phenotype, genotype, and the environment to which the mother is exposed competitively influence the offspring phenotype. Some maternal effects benefiting offspring fitness are referred to as adaptive maternal effects, which help offspring better adapt to the surrounding environment [4]. Accordingly, in humans, an increasing number of medical studies have been conducted to improve mother and infant health, and the findings suggest that maternal nutrition, immunity, and microbes may play roles in maternal effects [5]. However, the mechanisms of maternal effects require further elucidation so that they can be utilized effectively to improve human and animal health.

In our previous studies, to minimize the influence of maternal genotype and phenotype, Hy-Line^®^ Brown (commercial line) breeder hens (parental stock generation) were used as model animals. These breeder hens were selected because the parental stock has high genetic homozygosity and phenotypic consistency to further achieve stable production traits between their offspring (commercial stock generation) [6]. In addition, chick embryos are classic models for embryological studies [7]. Our previous studies have suggested that nutritional interventions, such as *β*-carotene, curcumin, allicin, and sodium butyrate, in laying breeder hens cause the offspring to exhibit increased growth performance and immunity in early life. This finding is potentially noted because the offspring exhibited improved intestinal immune establishment at the chick stage, which enhances intestinal development to further benefit growth [8]. Furthermore, microbial colonization is guided by innate immunity, and the microbiota further drives the maturation and development of gut-associated immune tissues as well as adaptive immunity. The continuous interaction between microbes and the host is associated with the intestinal immune development of chicks [9]. Adaptive maternal effects clearly exist, but how they are transferred and how they benefit offspring remain unclear.

Recent studies have discussed the microbial transmission from mother to chick, as well as the potential role of the maternal microbiota in chicks. Researchers have found that maternal gut microbes can be indirectly transferred to passerine chicks via nests and shape the early-life assembly of gut microbiota [10]. Moreover, maternal microbial transfer during hen raising can help chicks establish the gut microbiota in their early life and improve resistance to pathogen challenge [11]. However, most studies on maternal microbial transfer and its effects have focused on maternal gut microbiota and chicks, and these features should be classified as indirect microbial transfer.

The avian oviduct also presents a mass of microbiota [12]. In hens, the egg is formed as the yolk traverses the oviduct (consisting of the infundibulum, magnum, isthmus, shell gland, and vagina). A mature follicle descends from the ovary and then passes through the infundibulum for approximately 15–30 min. Fertilization occurs in the infundibulum if natural mating or artificial insemination has been conducted. Then, the follicle enters the magnum, where it is enveloped in egg white for 2–3 h. The eggshell membrane and eggshell are deposited in the isthmus and shell gland for 1.5–2 h and 18–20 h, respectively. Finally, the egg is laid from the vagina [13]. All substances are prepared well into the egg to support a new life generation, including the necessary genetic material, nutrition, and moisture, in addition to microbes and immune factors. After 21 days of incubation, a chick will break through the eggshell if ideal conditions are met. Based on the physiology of egg formation, we hypothesized that the vertical transfer of maternal oviduct microbes and immune factors could affect embryonic development to accomplish maternal effects.

Hence, this study aimed to reveal the role of maternal oviduct microbial transfer in maternal effects. In this study, we optimized previous experiments in an attempt to address these uncertainties. Briefly, laying breeder hens were given a nutritional intervention. We followed the process of egg formation in the maternal reproductive system and focused on the relationships between embryonic intestinal development, the embryonic intestinal transcriptome, and maternal microbial transfer.

## Methods

### Experimental model

Hy-Line^®^ Brown laying breeder hens (Rhode Island White, parental stock generation) aged 45 weeks were provided by the Changchun Academy of Agricultural Science, Changchun, China.

### Laying breeder hen feeding experiment and sampling

A flow diagram of the experimental design is shown in Additional file 1: Fig. S1. The experimental design in the current study was similar to our previous experimental design [8]. Briefly, a total of 486 laying breeder hens were randomly allocated to 2 groups: a control (CON) group and a group supplemented with *β*-carotene, curcumin, allicin, and sodium butyrate (the CCAB group). Each group included 3 replicate groups of 81 hens. The hens were kept in cages (120 × 60 × 60 cm^3^) equipped with 2 nipple drinkers and 1 feeder with 3 hens per cage. Semen was collected from Hy-Line^®^ Brown breeder cocks (Rhode Island Red) and injected into the hen oviducts. Artificial insemination was conducted once every 5 days in the afternoon. After a 1-week acclimation period, the hens in the CON group were fed a basal diet, whereas the hens in the CCAB group were fed the same basal diet supplemented with 60 mg/kg *β*-carotene, 250 mg/kg curcumin, 250 mg/kg allicin, and 500 mg/kg sodium butyrate (Shaanxi Kingreg Biotech Co., Ltd., Shaanxi, China) for 6 weeks. The detailed compositions of the basal diets (ingredients provided by WELLHOPE Group, Shenyang, China) given to the experimental hens are provided in Additional file 1: Table S1. All hens were allowed to eat and drink freely under the same growth, egg-laying, and feeding patterns.

At the end of the hen feeding experiment, we randomly selected 5 hens from each group for sampling following euthanasia. Serum samples were obtained via centrifugation of brachial venapuncture at 3000 rpm for 15 min and stored at − 80 °C for detection of serum immune factors. The central part of the magnum (approximately 2 cm) of each hen was fixed in 4% paraformaldehyde for immunohistochemistry-paraffin (IHC-P) assays. Magnum scraping samples were collected in germ-free tubes and stored in liquid nitrogen for magnum bacterial 16S rRNA gene sequencing and analysis. Ovary and magnum tissue samples were collected in RNase-free tubes and frozen at − 80 °C for further qPCR and Western blotting assays. Follicles were collected and stored at − 80 °C for determination of immune factors.

### Breeder egg hatching and offspring chick feeding experiments

A total of 900 breeder eggs from each group were collected and stored in a 16 °C refrigerator over the last 5 days during the hen experimental period for hatching. The eggs were hatched using an automatic incubator (Yiai Electronic Technology Co., Ltd., Bengbu, China) with 50–70% humidity (1–6 days at 60–70%, 7–14 days at 50–55%, and 14–21 days at 65–70%) at 37.8 °C with intermittent rotation. Embryo mortality was recorded during hatching. Considering that the maternal microbiota might represent an influencing factor, the eggs were not disinfected before hatching. The hen feeding (described above) and breeder egg hatching experiments were conducted at Changchun Academy of Agricultural Science, Changchun, China.

After the chicks hatched, we randomly selected 30 healthy male and 30 healthy female offspring chicks with similar body weights of approximately 38 g from each group for further separate feeding. All chicks were fed a single basal diet until 21 days of age. The offspring chicks from the hens in the CON and CCAB groups were considered the cCON and cCCAB groups, respectively. We recorded growth performance (including body weight and tibial length) in these groups every week to assess the repeatability of our previous studies and to assess whether the sex of the offspring was an influencing factor. The detailed compositions of the basal diets given to the experimental chicks and the chick-brooding management information are listed in Additional file 1: Tables S1 and S2.

### Embryonic development experiment and sampling

We transported 80 breeder eggs from each group to the laboratory simultaneously to ensure a controllable environment (including incubators predisinfection, daily disinfection of the clean room, and sterile water humidification) for hatching and to provide rapid and clean laboratory conditions (such as a clean room and sterile surgical instruments) for embryonic sampling. In addition, we assumed that the maternal microbiota might be a factor affecting offspring, so we did not disinfect the eggs. Before incubation, we quickly separated the yolks and egg whites of 6 eggs of each group in sterile plates and stored these samples at − 80 °C for the determination of immune factors in egg white and yolk and for egg white bacterial 16S rRNA gene sequencing and analysis. Then, the other breeder eggs were placed in incubators (one incubator per group) under 70–80% humidity at 37.8 °C with intermittent rotation. The embryos bred from the CON and CCAB groups were considered the eCON and eCCAB groups, respectively.

A total of 6 embryos from each group were randomly selected for sampling at embryonic day (E) 15, E17, E19, E20, and E21. Blood (200–500 μl) was collected from the yolk sac artery using capillary blood collection tubes, and serum samples were obtained by centrifugation at 4500 rpm for 15 min and stored at − 80 °C for the determination of immune factors. Amniotic fluid, yolk sac fluid, and embryonic egg white samples (0.5–1 ml) were drawn with sterile Pasteur pipettes and stored in sterile tubes at − 80 °C for determination of immune factors. Embryonic small intestinal and cecal samples were collected separately. Although the embryonic gut is too small (approximately 0.30 g at E15) to assess additional protein and gene expression following conventional methods, it is sufficient for RNA sequencing (RNA-seq) and histology analyses. We used anatomical features to identify the duodenum, jejunum, and ileum as described in our previous study [13]: the final intersection point of the duodenum and pancreas was considered the boundary between the duodenum and jejunum, and the yolk stalk served as the boundary between the jejunum and ileum. Then, the central part of each intestine was fixed in 4% paraformaldehyde to standardize for further morphological observations and IHC-P assays. Other tissues were then collected in RNase-free tubes and stored in liquid nitrogen for further RNA-seq analysis. Cecal tissues were stored in sterile tubes in liquid nitrogen for embryonic gut bacterial 16S rRNA gene sequencing analysis.

### ELISA

Commercial ELISA kits were used to quantify the levels of immune factors in the serum, magnums, and follicles of hens; yolks and albumins of eggs; and serum, albumins, yolk sac fluid, and amniotic fluid of embryos. Tissues (approximately 0.1 g) were ground in 1 ml of PBS and centrifuged at 3500 rpm for 15 min, and then, the protein levels in the supernatants were quantified using an Enhanced BCA Protein Assay Kit (Beyotime Biotechnology, P0009). The results are expressed as the mass/mg total protein. Small proteins were extracted from follicles, yolks, albumin, embryonic yolk sac fluid, and embryonic egg whites according to the method of Hamal et al. (2006) for ELISAs [14], and the concentrations are expressed as the mass/g follicle, yolk, or egg white. ELISA was conducted according to the manufacturer’s instructions with 3 technical repeats. The samples were diluted fivefold with 3 technical repeats. Chicken IgA, IgG, IgM, lysozyme (LYZ), and avian beta-defensins (AvBDs) ELISA kits were purchased from MeiMian Industrial Co., Ltd., Yancheng, China (cat. no. MM-0913O1, MM-0505O1, MM-0912O1, MM-34261O1, and MM-34120O1).

### Western blotting

Western blotting was used to detect the levels of LYZ and ovoinhibitor in the magnum. Total protein was extracted from each magnum tissue sample using RIPA lysis buffer (Beyotime Biotechnology, P0013B) supplemented with PSMF (Beyotime Biotechnology, ST506), quantified using an Enhanced BCA Protein Assay Kit (Beyotime Biotechnology, P0009), and heated at 100 °C for 5 min with 6 × Protein Loading Buffer (Transgen Biotech, DL101). Then, these samples (25 μg) were separated by electrophoresis on 12% SDS-PAGE gels (prepared with 30% Acr-Bis (29:1), 10% SDS, 1 M Tris–HCl (pH 6.8), 1.5 M Tris–HCl (pH 8.8), and APS; BOSTER Biological Technology, cat. no. AR1161, AR1164, AR1163, AR1162 and AR1166) and transferred to 0.2-μm PVDF membranes (Millipore, 6A6928). The membranes were blocked at 37 °C for 1 h in 5% dry milk dissolved in TBS-T buffer (1 × TBS, 0.1% Tween 20; TBS powder: Sangon Biotech, A500027-0004; Tween 20: Sigma‒Aldrich, P1379). Then, the blots were incubated overnight at 4 °C with primary antibodies diluted with 2% dry milk (BD, 232,100, 500 g) dissolved in TBS-T buffer. Next, the blots were incubated at 37 °C for 1 h with secondary antibodies diluted in 2% dry milk dissolved in TBS-T buffer. The membranes were rinsed with TBS-T buffer after each incubation step. Finally, the blots were detected with Immobilon Western Chemiluminescent HRP Substrate (Millipore, WBKLS0500) using a ChemiDoc MP Imaging System (Bio-Rad, 17,001,402). The following antibodies were used in the Western blot assays (with the dilution ratios indicated in parentheses): an unconjugated rabbit anti-chicken LYZ polyclonal antibody (1:2000, Abcam, ab391), an HRP-linked rabbit anti-chicken ovoinhibitor antibody (1:2000, Abcam, ab193507), an ACTB monoclonal antibody (1:5000, ABclonal Technology, AC026), an HRP-linked anti-mouse IgG antibody (1:2000, CST, 7076S), and an HRP-linked anti-rabbit IgG antibody (1:2000, CST, 7074S).

### IHC-P and observation of embryonic small intestinal morphology

IHC-P was used to investigate the expression and distribution of IgA, LYZ, and ovoinhibitor in the magnums of hens and of IgA and LYZ in the embryonic duodenum, jejunum, and ileum at E21. Briefly, 4% paraformaldehyde-fixed tissues were embedded in paraffin according to conventional methods. Sections (5 μm thickness) were cut onto gelatinized slides. The slides were deparaffinized, rehydrated, and placed in SSC buffer (BOSTER Biological Technology, AR0024) in a boiling water bath for 20 min for antigen retrieval. Then, we used commercial UltraSensitive™ Mouse and Rabbit SP IHC Kits (MXB Biotechnologies, KIT-9701 and KIT-9706) for endogenous peroxide elimination, blocking, biotin-linked secondary antibody incubation, and streptavidin incubation following the manufacturer’s instructions. The sections were incubated with primary antibodies in humidified chambers overnight at 4 °C after the blocking step. The sections were rinsed with PBS-T buffer (1 × PBS, 0.1% Tween 20) after each incubation. We used DAB MXB Biotechnologies, DAB-0031) and hematoxylin (Novon Scientific, China, SS0376) for chromogen/substrate and nuclear staining, respectively. Finally, after dehydration, clearing, and coverslipping, the sections were observed and photographed under a microscope (Olympus, IX71). The following antibodies (with dilution ratios in parentheses) were used in the IHC-P assays: a mouse anti-chicken IgA-UNLB antibody (1:200, SouthernBiotech, 8330–1) and an unconjugated rabbit anti-chicken LYZ polyclonal antibody (1:800, Abcam, ab391). The antibodies were diluted using commercial antibody diluent (BOSTER Biological Technology, AR1016).

Embryonic small intestinal morphological observations were conducted using hematoxylin and eosin (H&E) staining (Novon Scientific, SS0776). Paraformaldehyde-fixed and paraffin-embedded 5-μm sections of embryonic duodenum, jejunum, and ileum at E15, E17, E19, E20, and E21 were stained using hematoxylin and eosin (H&E) according to standard methods [15], and the sections were photographed under 200 × magnification using a microscope (Olympus, IX71). Finally, villus length and crypt depth were measured using microscope software (cellSens, version: 3.1). Measurements were performed as follows: villus height was determined from the villous tip to the villous-crypt junction, and crypt depth was measured from the opening to basing of the crypt.

### RNA extraction and qPCR

The mRNA expression of immunity-related antibacterial molecules and receptors in the magnum and ovary (*n* = 5) was determined by qPCR. Total RNA extraction, quality control, and quantification; cDNA synthesis; and qPCR were performed according to our previous description [9]. The primers are listed in Additional file 1: Table S3.

### Embryonic small intestinal RNA extraction and RNA-seq

We adopted RNA-seq to comprehensively investigate the gene expression in the small intestines of the offspring at E15 and E21. Duodenal, jejunal, and ileal tissues from the same individual were mixed to isolate total RNA using the TRIzol (Invitrogen, 15,596–026) method. The quantity and quality of the harvested RNA were measured using a NanoDrop spectrophotometer (Thermo Fisher Scientific, NC2000), agarose gel electrophoresis, and a Bioanalyzer 2100 System (Agilent, G2939BA). The quality control results are shown in Additional file 1: Table S4. Sequencing libraries were generated using a TruSeq RNA Sample Preparation Kit (Illumina, RS-122–2001). Then, 3 µg of RNA was used as input material for RNA sample preparation. Briefly, mRNA was purified from total RNA using poly-T oligo-attached magnetic beads. Fragmentation was performed using divalent cations under elevated temperature in an Illumina proprietary fragmentation buffer. First-strand cDNA was synthesized using random oligonucleotides and SuperScript II. Second-strand cDNA synthesis was subsequently performed using DNA Polymerase I and RNase H. The remaining overhangs were converted into blunt ends via exonuclease/polymerase activity, and the enzymes were removed. After adenylation of the 3′ ends of the DNA fragments, Illumina PE adapter oligonucleotides were ligated to prepare for hybridization. To select cDNA fragments with the preferred length of 200 bp, the library fragments were purified using an AMPure XP system (Beckman Coulter, A63881). DNA fragments with adaptor molecules ligated on both ends were selectively enriched using Illumina PCR Primer Cocktail in a 15-cycle PCR program. The products were purified using an AMPure XP system and quantified using an Agilent High-Sensitivity DNA assay (Agilent, 5067–1511) on the Agilent Bioanalyzer 2100 System. The sequencing libraries were then sequenced on an Illumina NovaSeq platform.

### DNA extraction and 16S rRNA sequencing

Total DNA was extracted from the magnum scraping samples, breeder-egg egg white samples, and embryonic cecal samples at E15 and E21 using a FastDNA® SPIN Kit (MP Biomedicals, 116540600). We used a NanoDrop spectrophotometer (Thermo Fisher Scientific, NC2000) and 1.2% agarose gel electrophoresis to measure the quantity and quality of the harvested DNA, respectively. Briefly, PCR amplification of the V3 to V4 region of the 16S rRNA gene was performed using the forward primer 338F and the reverse primer 806R with sample-specific 7-bp barcodes. Model microbes and sterile water were used as positive and negative controls, respectively, from DNA extraction to sequencing. The PCR system contained 5 μl of Q5 reaction buffer (5 ×), 5 μl of Q5 High-Fidelity GC buffer (5 ×), 0.25 μl of High-Fidelity DNA Polymerase (5 U/μl), 2 μl (2.5 mM) of dNTPs, 1 μl (10 µM) of each forward and reverse primer, 2 μl of DNA template, and 8.75 μl of ddH_2_O. The thermal cycling program consisted of an initial denaturation step at 98 °C for 2 min followed by 25 cycles of denaturation at 98 °C for 15 s, annealing at 55 °C for 30 s, and extension at 72 °C for 30 s with a final extension step of 5 min at 72 °C. The electrophoresis results of the PCR amplification products are shown in Additional file 1: Fig. S2. The harvested PCR amplicons were purified using an Agencourt AMPure XP Kit (Beckman Coulter, A63881) and quantified using a Quant-iT™ PicoGreen™ dsDNA Assay Kit (Invitrogen, P7589). The concentrations of purified PCR amplicons are listed in Additional file 1: Table S5. After harvest, purification, and quantification, the PCR amplicons were pooled in equal amounts for paired-end 2 × 300-bp sequencing on an Illumina NovaSeq platform.

### Bioinformatics analyses of RNA-seq data

RNA-seq samples were sequenced on the platform to obtain image files, which were transformed by the platform software into FASTQ format (raw data). The sequencing data contained a number of adaptors and low-quality reads, so we used cutadapt software to filter the sequencing data and obtain high-quality sequences (clean data) for further analysis [16]. The quality control results for the filtered reads are listed in Additional file 1: Table S6. For data mapping analysis, a reference genome and gene annotation files were downloaded from Ensembl (http://asia.ensembl.org/Gallus_gallus/Info/Index). The filtered reads were mapped to the reference genome using HISAT2 software, and the default mismatch parameter (no more than 2 mismatches) was used. The mapping summary (Additional file 1: Table S7) and gene coverage (Additional file 1: Fig. S3) results were used for mapping quality control. We used HTSeq (version: 0.9.1) statistics to compare the read count values for the genes as the original expression levels of the genes according to the “union” strategy and then used the fragments per kilobase per million mapped reads (FPKM) values to standardize the expression [17]. Next, we used the DESeq R package (DESeq version: 1.30.0, R version: 4.0.3) to perform principal component analysis (PCA) and screened the differentially expressed genes (DEGs) with criteria of |log2FoldChange|> 1 and *P* adj < 0.05. Volcano maps of the DEGs were generated using the ggplot2 R package (version: 3.3.3). Next, we mapped all the genes to terms in the Gene Ontology (GO) database (http://geneontology.org) and calculated the number of DEGs enriched for each term. Based on the whole genome, terms with significant (*P* adj < 0.05) enrichment of DEGs were calculated by hypergeometric distribution.

### Bioinformatics analyses of 16S rRNA sequencing data

The QIIME2 (version: 2019.4) pipeline and R packages were employed for bioinformatics analyses of the 16S rRNA sequencing data [18]. Raw reads were demultiplexed using the q2(QIIME2)-demux plug-in followed by primer cutting with the q2-cutadapt plug-in [16]. The sequences were then quality filtered, denoised, and merged, and chimeras were removed using the q2-dada2 plug-in [19]. Non-singleton amplicon sequence variants (ASVs) were aligned with MAFFT via the q2-alignment plug-in [20] and used to construct a phylogeny with FastTree2 via the q2-phylogeny plug-in [21]. The ASVs were annotated with taxa using the classify-sklearn naïve Bayes taxonomy classifier in the q2-feature-classifier plug-in [22] against Greengenes (version: 13_8) 99% OTU reference sequences [23]. The possible reagent contaminant-related taxa were filtered by referencing the opinion of Eisenhofer et al. (2019) [24], negative sequencing and related studies that reported putative contaminants [25], and chicken oviduct microbiota [12, 26–28]. The unique and shared ASVs between groups were calculated using the VennDiagram R package (version: 1.7.0). A Bray‒Curtis dissimilarity matrix was calculated to investigate nonmetric multidimensional scaling (NMDS) for beta-diversity analysis via the q2-diversity plug-in. Then, the data were visualized via the vegan R package (version: 2.5–7). The SourceTracker R package (version: 1.0.1) was used to estimate the source magnum proportions of microbes in egg white and E15 and E21 embryonic gut with 100 draws from Gibbs sampling [29]. We used linear discriminant analysis effect size (LEfSe) with Kruskal‒Wallis and Wilcoxon tests and a one-against-all strategy to detect significantly enriched (log_10_(LDA score) > 2, *P* < 0.05) taxa from the phylum to species levels between groups by submitting a formatted ASV table to the Galaxy online analysis platform [30].

### Statistical analyses and visualization

We used the FPKM values of significantly upregulated DEGs [31] and the relative abundance values of significantly enriched taxa in each terminal clade in the LEfSe cladogram to perform Spearman correlation analysis using IBM SPSS Statistics 23 (IBM, version: 23.0.0.0) software. The results were visualized as heatmaps using Prism 8 (GraphPad, version: 8.4.3) software. Similarly, the Spearman correlations between the FPKM values of significantly upregulated DEGs and the intestinal morphological indices consisting of villus height and crypt depth were analyzed and visualized. Student’s *t*-test was also performed using SPSS software to detect significant differences between groups. The results were visualized with Prism 8 software and are expressed as the means ± SEMs. A value of *P* < 0.05 was considered to indicate statistical significance, which is denoted in the figures as follows: *ns P* ≥ 0.05, *P < 0.05, and **P < 0.01.

## Results

### Maternal nutritional intervention benefits maternal immunity, egg hatching, and offspring growth

**Fig. 1:**
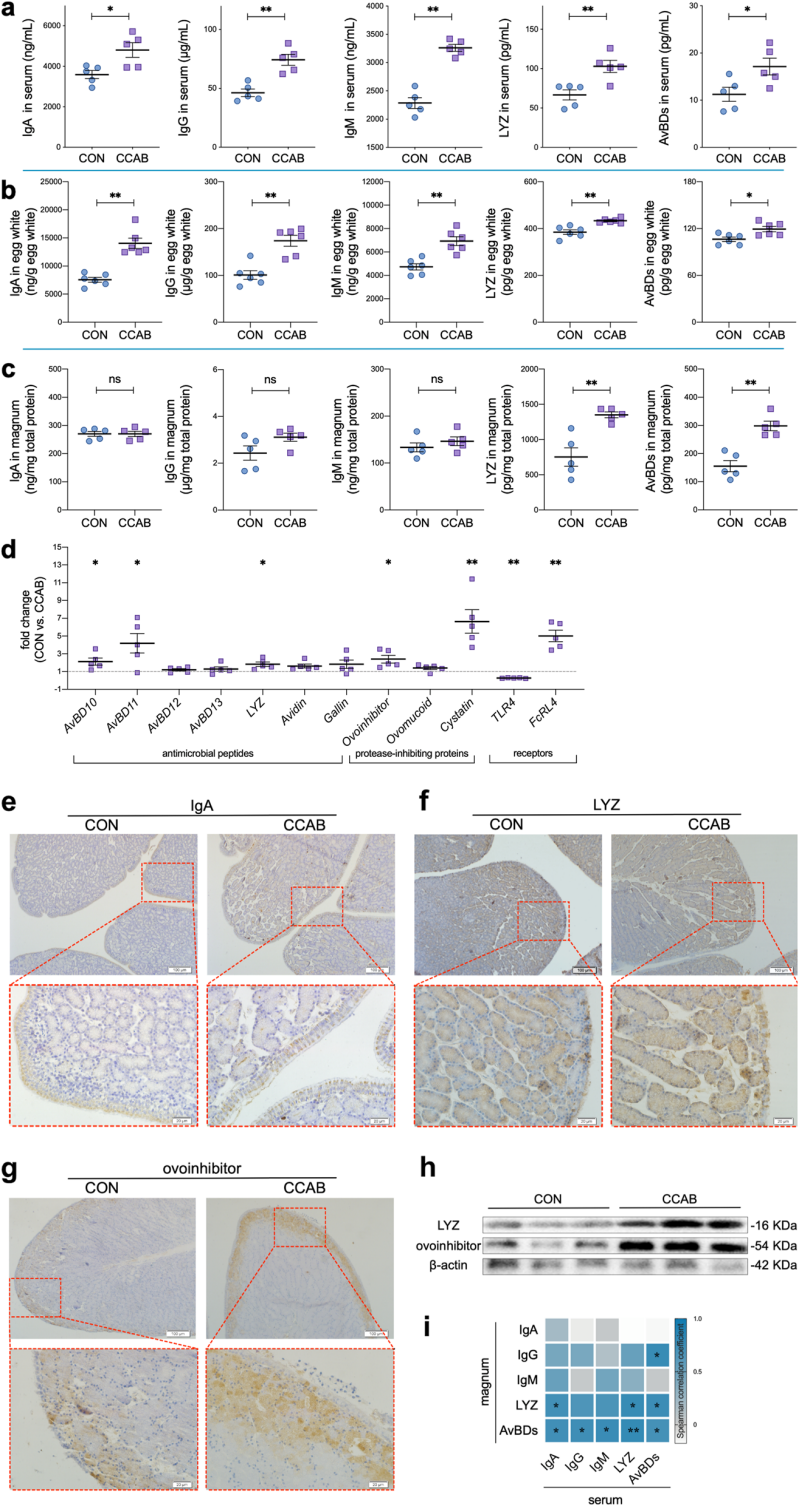
Transfer of immune factors into egg whites depends on maternal levels. **a** Serum IgA, IgG, IgM, LYZ, and AvBDs levels in hens as determined by ELISA. *n* = 5 hens. **b** IgA, IgG, IgM, LYZ, and AvBDs levels in the egg whites of breeder eggs as determined by ELISA. *n* = 6 eggs. **c** IgA, IgG, IgM, LYZ, and AvBDs levels in the magnums of hens as determined by ELISA. *n* = 5 hens. **d** Expression of genes encoding antimicrobial peptides, protease-inhibiting proteins, and receptors in the magnums of hens as determined by qPCR. *n* = 5 hens. **e**–**g** Representative images of the expression of IgA, LYZ, and ovoinhibitor in the magnums of hens as indicated by IHC-P. Bars = 100 μm (top row) and 20 μm (bottom row). **h** LYZ and ovoinhibitor expression in the magnum as determined by Western blotting. *n* = 3 hens. **i** Spearman correlations of immune factors between the serum and magnum in hens. *P < 0.05 and **P < 0.01, *n* = 10. Data are shown as the means ± SEMs (**a**–**d**). Student’s *t*-test was conducted. *ns*, *P* ≥ 0.05, *P < 0.05, and **P < 0.01

In this study, we supplemented laying breeder hen diets with *β*-carotene, curcumin, allicin, and sodium butyrate (CCAB) to improve hen immunity and further observed the growth performance of the offspring chicks in early life (1–21 days of age). Serum IgA, IgG (IgY), IgM, lysozyme (LYZ), and avian beta-defensins (AvBDs) levels in hens were significantly higher in the CCAB group compared with the control (CON) group (Fig. 1a). Moreover, the CCAB offspring chicks maintained better growth performance than the CON offspring chicks (Additional file 1: Fig. S4a). The embryonic mortality of breeder eggs was lower in the CCAB group than in the CON group (Additional file 1: Fig. S4b).

### Transfer of immune factors into eggs depends on maternal levels

**Fig. 2:**
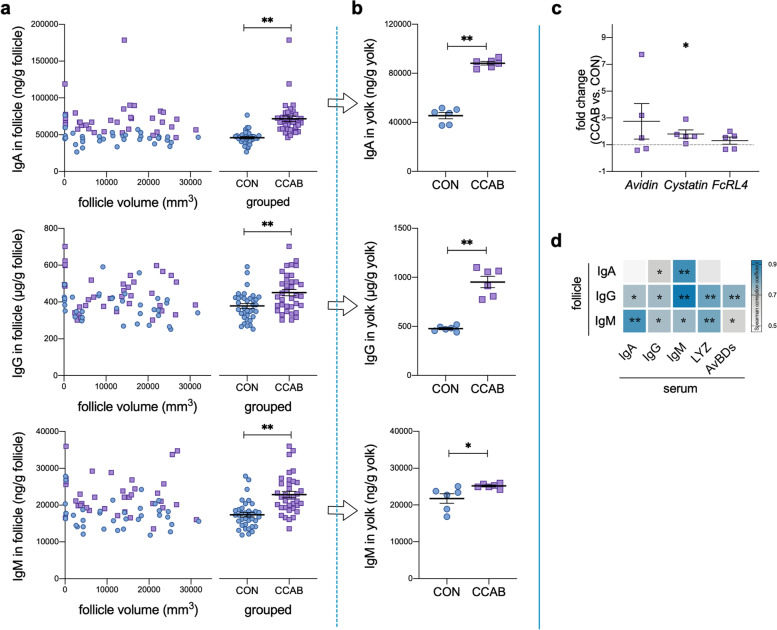
Transfer of immune factors into yolks depends on maternal levels. **a** IgA, IgG, and IgM levels in follicles as determined by ELISA. *n* = 36 follicles with different sizes from 5 hens.** b** IgA, IgG, and IgM levels in breeder-egg yolks as determined by ELISA. *n* = 6 eggs. **c** Gene expression of *Avidin*, *Cystatin*, and *FcRL4* in the ovaries of hens as determined by qPCR. *n* = 5 hens. **d** Spearman correlations of immune factors between the follicles and serum of hens. *P < 0.05 and **P < 0.01, *n* = 10. Data are shown as means ± SEMs (**a**–**c**). Student’s *t*-test was conducted. *ns*, *P* ≥ 0.05, *P < 0.05, and **P < 0.01

We investigated the levels of immune factors in egg whites and yolks of hen eggs. The levels of IgA, IgG, IgM, LYZ, and AvBDs in egg white (Fig. 1b) and the levels of IgA, IgG, and IgM in yolk (Fig. 2b) were significantly increased in the CCAB group.

Next, we examined the hen magnum, follicle, and ovary, as egg white is synthesized and secreted in the magnum, whereas the follicle develops into yolk in the ovary. In the magnum, the levels of LYZ and AvBDs were significantly higher in the CCAB group than in the CON group, whereas IgA, IgG, and IgM levels did not differ between the groups (Fig. 1c). The *FcRL4* gene was upregulated significantly in the magnum in the CCAB group (Fig. 1d) as were the antimicrobial peptide genes *AvBD10*, *AvBD11*, and *LYZ* and the protease-inhibiting protein genes *ovoinhibitor* and *cystatin*; in contrast, the receptor gene *TLR4* was significantly downregulated (Fig. 1d). Additionally, we investigated the protein expression levels of these differentially expressed genes using the limited commercial antibodies available for chickens. The results of IHC-P assays for IgA, LYZ, and ovoinhibitor (Fig. 1 e, f, and g) and the results of Western blotting assays for LYZ and ovoinhibitor (Fig. 1h) coincided with the results of gene expression analyses and ELISAs. Moreover, we investigated the correlations of these immune factors between the serum and magnum (Fig. 1i). Notably, the levels of LYZ and AvBDs in the magnum were positively correlated ($\overline{r }$ = 0.738) with the levels of IgA, IgG, IgM, LYZ, and AvBDs in serum.

We also assessed the levels of IgA, IgG, and IgM in follicles of different sizes and found that they remained constant regardless of the follicle size (Fig. 2a). The levels of IgA, IgG, and IgM during follicular development were significantly increased in the CCAB group (Fig. 2a). In addition, the levels of these immunoglobulins in follicles were positively correlated ($\overline{r }$ = 0.724) with the levels of IgA, IgG, IgM, LYZ, and AvBDs in serum (Fig. 2d). The *cystatin* gene was significantly upregulated in the ovary (Fig. 2c).

### Promotion of offspring intestinal development begins in the embryonic period

**Fig. 3:**
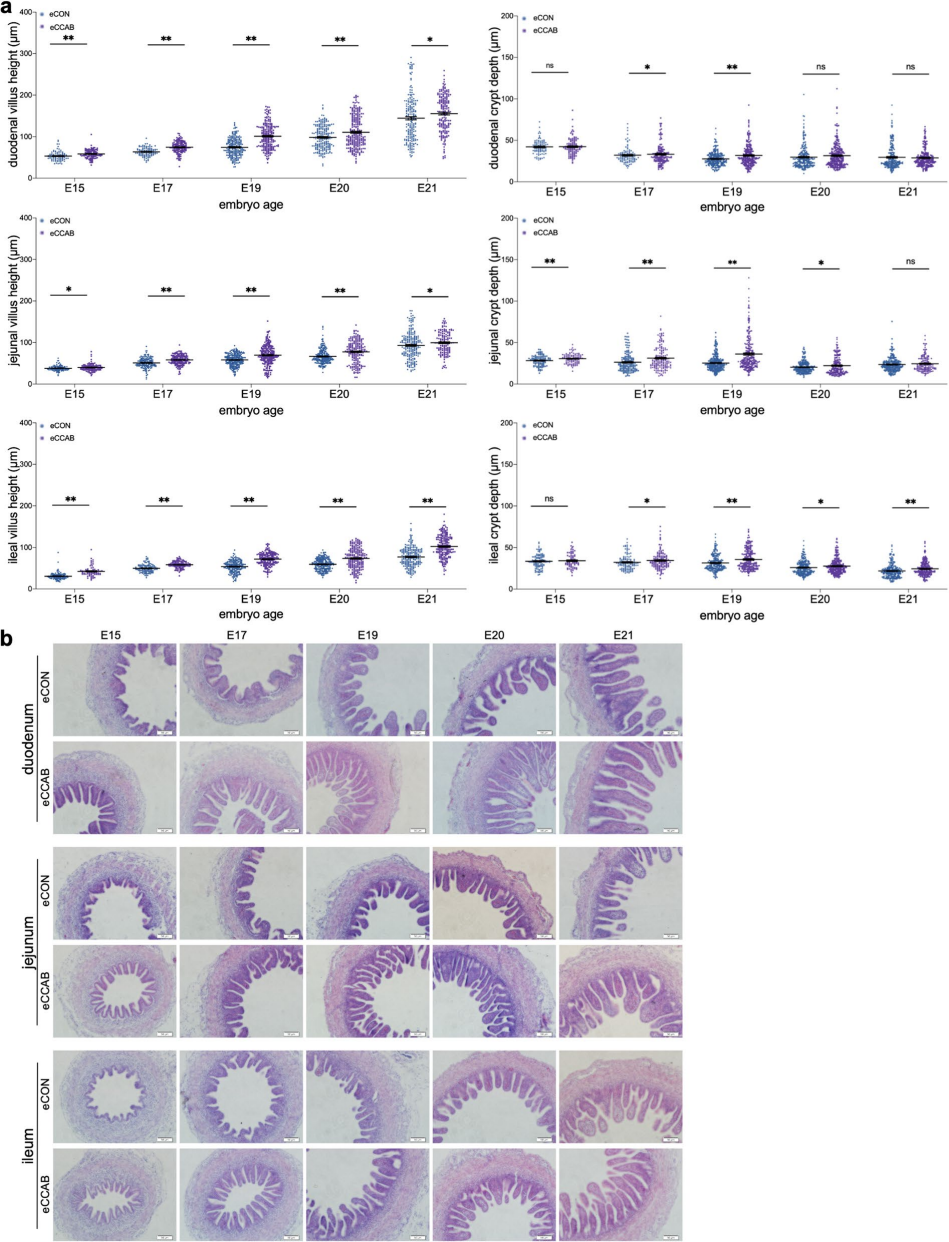
Promotion of offspring intestinal development begins in the embryonic period. **a** Villus height (left) and crypt depth (right) in the embryonic duodenum, jejunum, and ileum at E15, E17, E19, E20, and E21. In total, 71 ≤ *n* ≤ 228 villi and crypts were obtained from 6 embryos in each group. Data are shown as the means ± SEMs. Student’s *t*-test was conducted. *ns P* ≥ 0.05, *P < 0.05, and **P < 0.01.** b** Representative morphological images of H&E-stained, formalin-fixed, and paraffin-embedded 5-μm sections of embryonic intestines at E15, E17, E19, E20, and E21. Bar = 50 μm

To evaluate embryonic small intestinal development, we measured the villus height and crypt depth in the embryonic duodenum, jejunum, and ileum at embryonic day (E) 15, E17, E19, E20, and E21. The villus heights in the duodenum, jejunum, and ileum at E15, E17, E19, E20, and E21 were significantly greater in the CCAB embryo (eCCAB) group compared with the control embryo (eCON) group (Fig. 3a). The crypt depth in the duodenum at E19; in the jejunum at E15, E17, E19, and E20; and in the ileum at E17, E19, E20, and E21 was significantly greater in the eCCAB group than in the eCON group. Representative morphological images are shown in Fig. 3b. Moreover, we also assessed the levels of IgA, IgG, IgM, LYZ, and AvBDs in embryonic serum and egg white; the levels of IgA, IgA, and IgM in embryonic yolk sac fluid; and the levels of LYZ and AvBDs in amniotic fluid for embryos at different ages. However, no significant differences were noted between groups (Additional file 1: Fig. S5).

### Maternal microbe transfer to the embryonic gut from the magnum to the egg white

**Fig. 4:**
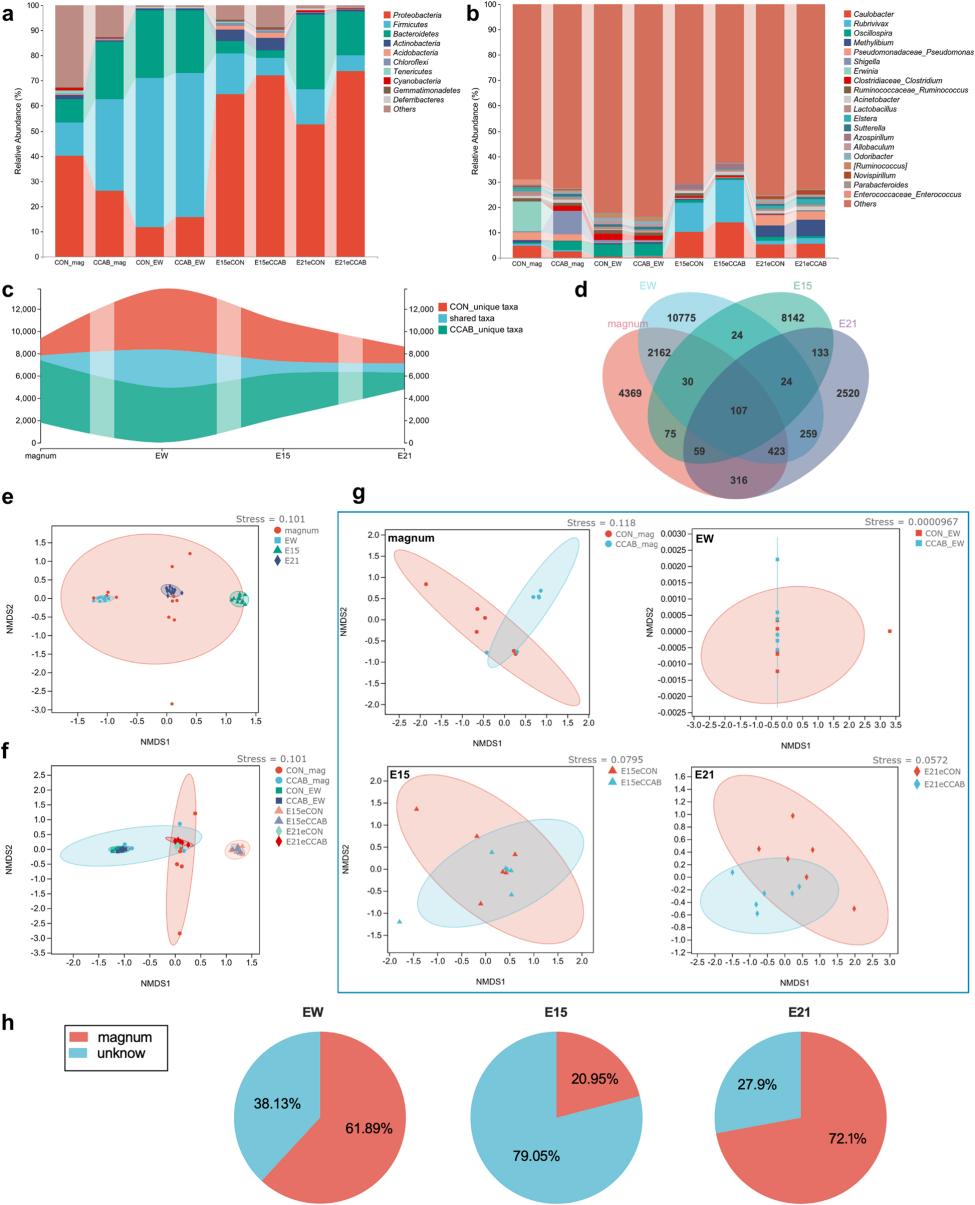
Maternal microbes transfer to the embryonic gut from the magnum to the egg white. Microbial composition at the phylum (**a**) and genus (**b**) levels, unique and shared taxa between groups (**c**), and between sample types (**d**) in the magnum (abbreviated as mag), egg white (abbreviated as EW), and E15 and E21 embryonic gut, *n* = 6 hens/eggs/embryos. **e** Beta diversity of microbes in the magnum, egg white, and E15 and E21 embryonic gut (*n* = 12). **f** Overview of the beta diversity of microbes between groups in the magnum, egg white, and E15 and E21 embryonic gut (*n* = 6). **g** Beta diversity of microbes between groups specifically in the magnum, egg white, and E15 and E21 embryonic gut (*n* = 6). Ellipses denote 95% confidence intervals. **h** Source of the magnum (*n* = 12) proportions of microbes in the egg white and E15 and E21 embryonic gut estimated using SourceTracker with 100 Gibbs draws

We investigated the microbial communities in the magnum mucosa, egg white, and embryonic gut in each group at E15 and E21 using 16S rRNA sequencing. The phyla *Proteobacteria*, *Firmicutes*, *Bacteroidetes*, and *Actinobacteria* (Fig. 4a) and the genera *Caulobacter*, *Rubrivivax*, *Oscillospira*, *Methylibum*, *Pseudomonas*, *Shigella*, and *Erwinia* (Fig. 4b) exhibited large relative abundances and were the dominant taxa in the magnum mucosa, egg white, and embryonic gut simultaneously (the sample levels are shown in Additional file 1: Fig. S6). A total of 420, 3397, 1073, and 839 taxa were shared between groups respectively in magnum, egg white, and E15 and E21 embryonic gut (Fig. 4c), and 107 taxa were shared among the magnum, egg white, and E15 and E21 embryonic gut (Fig. 4d, the detailed list of shared taxa is provided in Additional file 1: Table S8). Moreover, the microbial beta diversities of the egg white and embryonic gut at E15 and E21 were clustered with that of the magnum mucosa (Fig. 4e); however, differences emerged between groups (Fig. 4 f and g). SourceTracker results showed that 61.89%, 20.95%, and 72.1% of microbes in egg white and E15 and E21 embryonic guts, respectively, were sourced from magnum (Fig. 4h). Next, we detected significantly differentially enriched taxa (log_10_(LDA score) > 2, *P* < 0.05) from the phylum to species levels between groups using LEfSe with Kruskal‒Wallis and Wilcoxon tests and a one-against-all strategy. LEfSe results indicated that 57/62 (the denominator means the total number of enriched taxa), 10/15, 3/8, and 21/41 taxa at multiple taxonomic levels were significantly enriched in the magnum mucosa in the CCAB-group hens, the egg white in CCAB-group eggs, the gut in eCCAB-group E15 embryos, and the gut in eCCAB-group E21 embryos, respectively, as shown in Additional file 1: Fig. S7. Notably, *Bifidobacterium animalis* was significantly enriched in the magnum in the CCAB group, whereas *Campylobacterales* and *Helicobacteraceae* were significantly enriched in the gut in eCON-group embryos at E21.

### Offspring embryonic intestinal transcriptome shifts are related to development and immunity

**Fig. 5:**
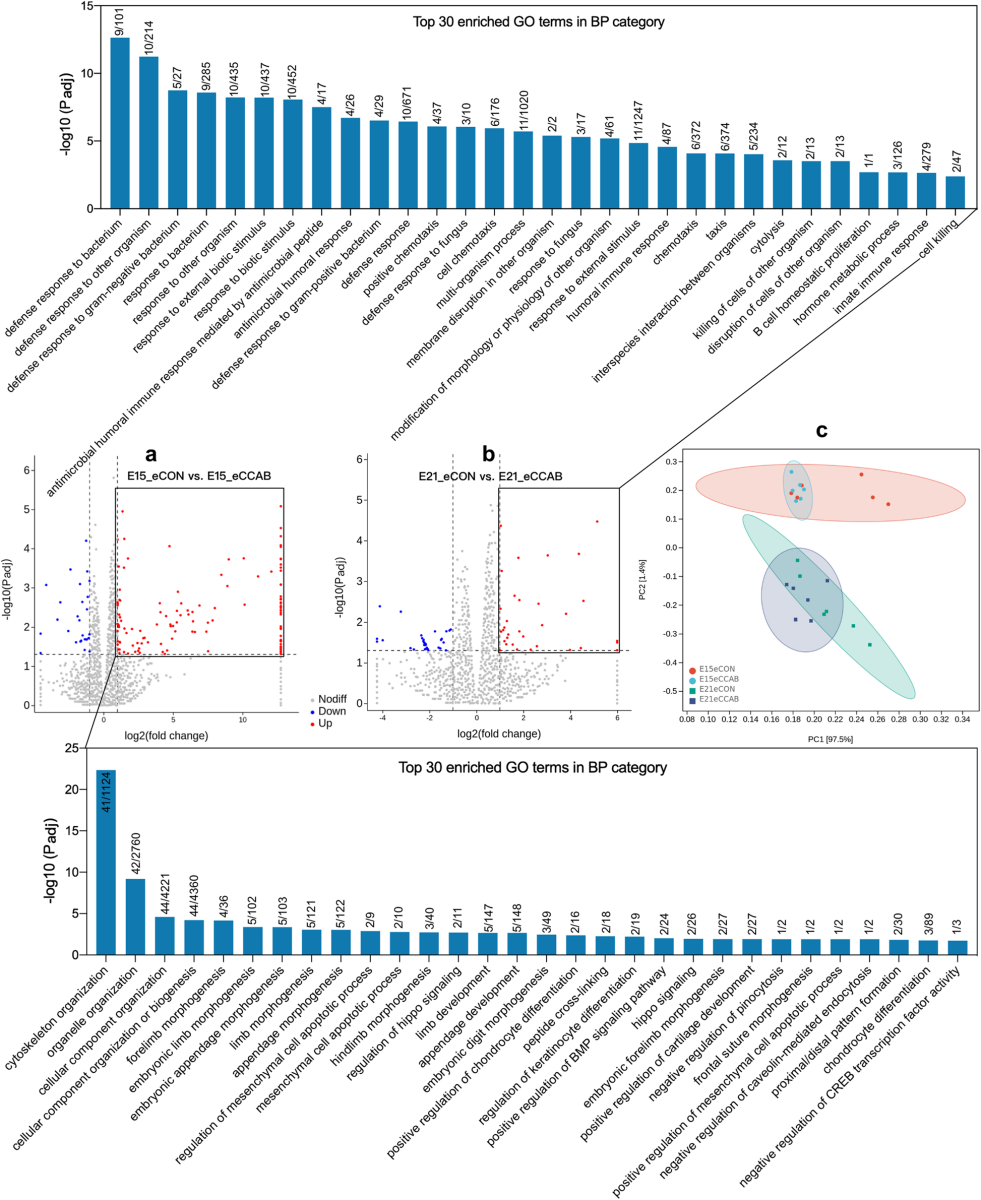
Offspring embryonic intestinal transcriptome shifts are related to development and immunity. Top 30 enriched GO terms with rich factors (the DEG number/total gene number in the term) in the BP category for upregulated DEGs in the embryonic intestinal transcriptome at E15 (**a**) and E21 (**b**). *n* = 6 embryos. **c** PCA of the embryonic intestinal transcriptome at E15 and E21. *n* = 6 embryos

We performed RNA-seq of embryonic small intestines at E15 and E21. PCA illustrated transcriptional changes between E15 and E21. Intestinal transcription also differed between groups (Fig. 5c).

Therefore, we next calculated the differentially expressed genes (DEGs, |log_2_FoldChange|> 1, *P* adj < 0.05) in embryonic small intestines at E15 and E21 between groups and performed Gene Ontology (GO) enrichment analysis on these DEGs. At E15 (Fig. 5a), a total of 125 upregulated DEGs and 27 downregulated DEGs were noted in the eCCAB group compared with the eCON group. The top 5 significantly enriched GO terms with a rich factor (DEG number/total gene number in the term) in the biological process (BP) category for these upregulated DEGs were as follows: cytoskeleton organization (41/1124), organelle organization (42/2760), cellular component organization (44/4221), cellular component organization or biogenesis (44/4360), and forelimb morphogenesis (4/36). At E21 (Fig. 5b), 33 upregulated DEGs and 34 downregulated DEGs were noted in the eCCAB group compared with the eCON group. The top 5 significantly enriched GO terms with a rich factor in the BP category for the upregulated DEGs were as follows: defense response to bacterium (9/101), defense response to other organism (10/214), defense response to gram-negative bacterium (5/27), response to bacterium (9/285), and response to other organism (10/435). Furthermore, the top 30 significantly enriched GO terms in the BP category for the upregulated DEGs are shown in Fig. 5, and the detailed results regarding the upregulated DEGs and the significantly enriched GO terms in the BP category are listed in Additional file 2.

**Fig. 6:**
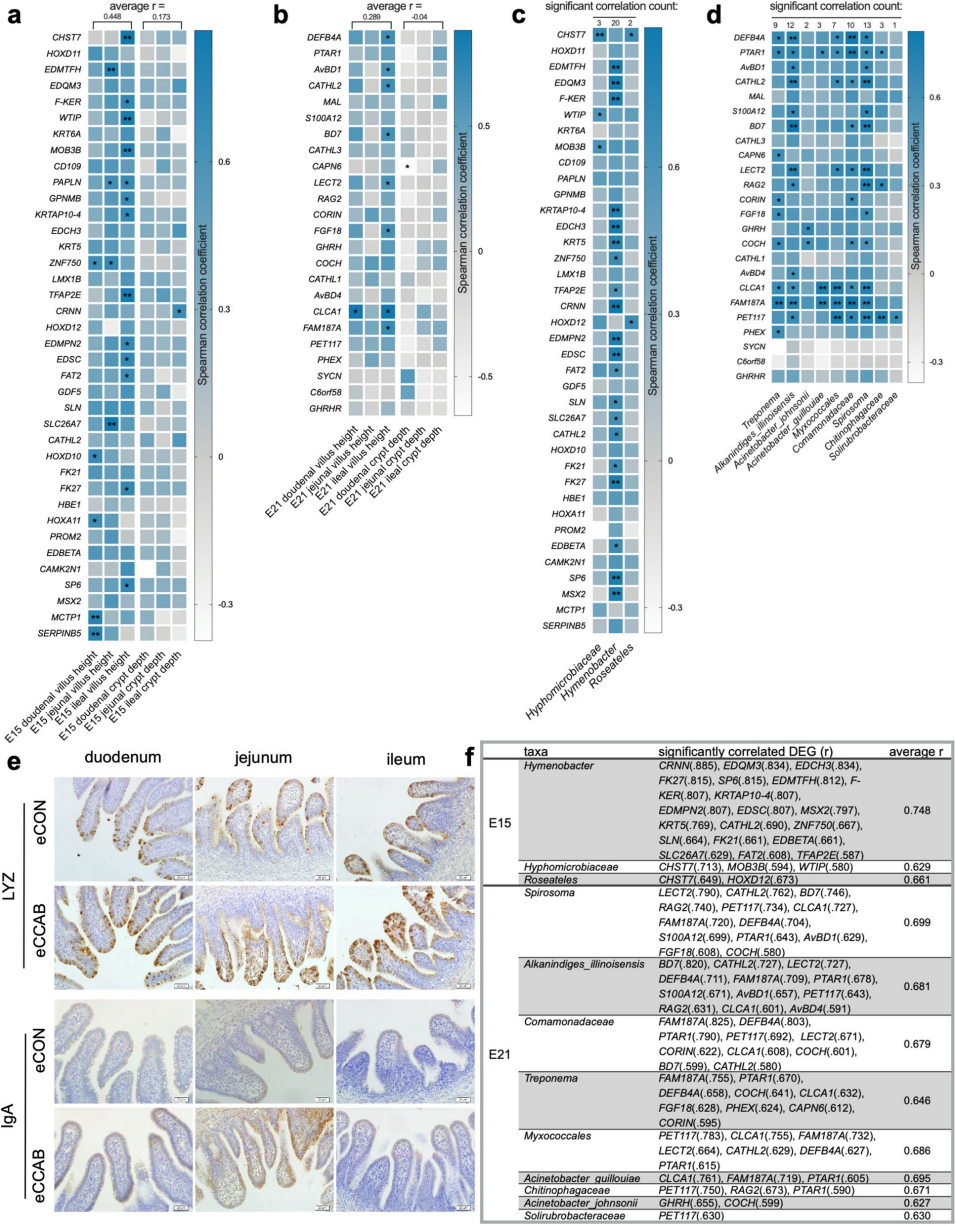
The embryonic gut microbiota is correlated with the intestinal transcriptome and intestinal development. Spearman correlations between upregulated DEGs and morphological indices at E15 (**a**) and E21 (**b**). *P < 0.05 and **P < 0.01, *n* = 12. Spearman correlations between upregulated DEGs and significantly enriched taxa at E15 (**c**) and E21 (**d**), which are summarized and ranked in (**f**). *P < 0.05 and **P < 0.01, *n* = 12. **e** Representative photographs of sections subjected to IHC-P showing embryonic intestinal mucosal immunity at E21. Bar = 20 μm

Notably, most of the top 30 enriched GO terms were associated with development and immunity at E15 and E21. Thus, we further used IHC-P to investigate small intestinal mucosal immunity at E21 (Fig. 6e). The levels of LYZ in the duodenum, jejunum, and ileum were higher in the eCCAB group compared with the eCON group, and the level of IgA in the jejunum was also higher in the eCCAB group than in the CON group.

### The embryonic gut microbiota is correlated with the intestinal transcriptome and intestinal development

We first performed Spearman correlation analysis between the expression of the upregulated DEGs and intestinal morphological indices (villus height and crypt depth) (Fig. 6 a and b). Generally, at E15, the expression of most of the upregulated DEGs was more positively correlated with villus height ($\overline{r }$ = 0.448) compared with crypt depth ($\overline{r }$ = 0.173); similar findings were obtained at E21 ($\overline{r }$ = 0.289 between the upregulated DEGs and villus height, $\overline{r }$ =  − 0.040 between the upregulated DEGs and crypt depth). Notably, the expression of the genes *CHST7*, *EDMTFH*, *F-KER*, *WTIP*, *MOB3B*, *PAPLN*, *GPNMB*, *KRTAP10-4*, *ZNF750*, *TFAP2E*, *EDMPN2*, *EDSC*, *FAT2*, *SLC26A7*, *HOXD10*, *FK27*, *HOXA11*, *SP6*, *MCTP1*, and *SERPINB5* was significantly positively correlated (*r* ≥ 0.636, *P* < 0.05) with villus height in the duodenum and/or jejunum and/or ileum at E15. The expression of the genes *BEFB4A*, *AvBD1*, *CATHL2*, *BD7*, *LECT2*, *FGF18*, *CLCA1*, and *FAM187A* was significantly positively correlated (*r* ≥ 0.587, *P* < 0.05) with villus height in the duodenum and/or ileum at E21.

We next conducted Spearman correlation analysis between the expression of the upregulated DEGs and the abundances of the significantly enriched taxa. At E15 (Fig. 6c), 20, 3, and 2 upregulated DEGs were significantly (*P* < 0.05) positively correlated with the abundances of *Hymenobacter* ($\overline{r }$ = 0.748), *Hyphomicrobiaceae* ($\overline{r }$ = 0.629), and *Roseateles* ($\overline{r }$ = 0.661), as summarized in Fig. 6f. At E21 (Fig. 6d), the top 3 correlated taxa (ranked by the number of significantly correlated DEGs) included *Spirosoma* ($\overline{r }$ = 0.699), *Alkanindiges illinoisensis* ($\overline{r }$ = 0.681), and *Comamonadaceae* ($\overline{r }$ = 0.679) with 13, 12, and 10 DEGs, respectively. The detailed results are summarized in Fig. 6f. Notably, most of the taxa identified by correlation analysis (except *Hymenobacter*, *Treponema*, *Myxococcales*, and *Solirubrobacteraceae*) were also shared among the magnum, egg white, and E15 and E21 embryonic gut (Additional file 1: Table S8).

## Discussion

In this study, we obtained results similar to those from our previous analyses [8, 9] regarding increased levels of immunoglobulins (IgA, IgG, and IgM) and innate immunity-related factors (LYZ and AvBDs) in the serum of hens and improved growth performance of their offspring chicks in early life. Consequently, our study shows that nutritional intervention benefits the immunity of hens and the growth performance of their offspring in early life. However, in birds, how are mothers’ immune advantages transferred to and/or benefit their offspring? What role does maternal immune and microbial transfer play in maternal effects?

Mammals exhibit the most complex reproductive behaviors and physiological components/mechanisms, such as the placenta, viviparous reproductive style, and breastfeeding, which optimize the survival rates of their offspring in complex environments [32]. Birds are also higher vertebrates that are widely distributed in nature. Some birds can be domesticated, bred, and industrialized to supply eggs and meat for humans; these uses are inseparable from their relatively complex reproductive behaviors and physiologies [33]. Intriguingly, eggs are continuously exposed to the environment and face the challenge of exposure to microbes and pathogenic agents during natural incubation; however, most eggs are still able to hatch successfully based on multiple defense mechanisms, including physical, chemical, and immune barriers that operate in eggs before and during incubation [34]. Before incubation, the eggshell and eggshell membrane, which are coated with many antimicrobial peptides, form the first physical and immune barriers [35]. Egg white contains numerous ovomucins, antimicrobial proteins, and protease-inhibiting proteins [36], such as the LYZ, AvBDs, and ovoinhibitor investigated in this study; these proteins create a physical and immune barrier. The vitelline membrane, which has a highly fibrous structure in which a series of antimicrobial proteins are deposited, is the last physical and immune barrier [37]. During incubation, new defense structures are formed. With embryonic development, the allantoic sac is filled with fluid to provide an acidic antibacterial environment, and it grows to cover the interior of the whole eggshell [38]. Then, the chorioallantoic membrane serves as a new physical, chemical, and immune barrier next to the eggshell and eggshell membrane that features immune cells and can implement an inflammatory response [39]. The amniotic membrane and amniotic fluid contain a series of antimicrobial peptides that are transferred from egg white [40] and become the last physical and immune barriers of the embryo. In mammals, the embryo receives a constant supply of nutrients from the mother and is well protected in the mother’s uterus. However, in birds, all substances needed to support the development of the offspring must be completely transferred from the mother into the egg before laying. Theoretically, maternal immunity and health in birds during the laying period should affect offspring embryonic safety and development.

In our study, we found that the embryos from the CCAB group exhibited a decreased mortality rate, and that the levels of immune factors were increased in the egg whites and yolks of the CCAB-group eggs (Figs. 1b and 2b). We traced the deposition of these immune factors back to the magnum, follicle, and ovary because egg white is synthesized and secreted by the magnum, whereas the yolk develops from the follicle in the ovary. We found that the expression of innate immune factors (LYZ and AvBDs), ovoinhibitor, and the *FcRL4* gene was increased significantly in the magnum in the CCAB group (Fig. 1 c, d, f, g, and h). Immunoglobulins were heavily deposited into follicles during vitellogenesis (Fig. 2a), and the *cystatin* gene was highly upregulated in the ovary (Fig. 2c). The amount of immunoglobulins transferred from the hen into the yolk and egg white is proportionally related to their maternal serum concentrations, and the immunoglobulins are transported via the Fcγ receptor [14, 41]. Maternal IgG is transported to the fetus across the placental barrier by the Fc receptor in mammals, which causes humoral immune levels in neonates similar to those in mothers [42]. In addition, multiple innate immune peptides are expressed in the oviduct [43]. These maternal immune factors provide protection for the embryo [44]. Therefore, our data (Figs. 1 and 2) provide evidence that the increased levels of immune factors in the eggs of the CCAB group resulted from increased deposition during the periods of egg white and yolk formation in the maternal reproductive system. We suggest that immune factors are vertically transferred into eggs from the reproductive system of the mother in a manner dependent on maternal levels (based on maternal immunity) to provide protection for embryonic development. Specifically, the transfer of higher levels of maternal immune factors from hens with stronger immunity might increase the chance that the chicks will hatch.

We further observed embryonic small intestinal morphology and found that the small intestinal development of the eCCAB group exhibited more advanced morphogenesis than that of the eCON group (Fig. 3). What factors caused the differences in embryonic intestinal development? In addition to the added protection provided by elevated levels of immune factors transferred from the mother, the findings of Ding et al. (2017) led us to hypothesize that the maternal microbiota may represent a factor [45]. Ding et al. reported that maternal microbial colonization occurs during embryonic development and speculated that microbes that are present in eggs were transferred from the oviduct during the process of egg formation (although they correlated the maternal fecal microbiota with the embryonic microbiota). Lee et al. suggested that egg white is “seeded” with microbes from the maternal oviduct [28]. Based on these previous findings, we investigated the microbial composition, beta-diversity, and source magnum proportion in the magnum, egg white, and embryonic gut and found that the microbial composition and beta diversity in the egg white and embryonic gut were highly similar to those in the maternal magnum, and large proportions of microbes in the egg white and embryonic gut were sourced from the maternal magnum (Fig. 4). Therefore, our data also support the idea that microbes in the magnum are transferred into egg white for colonization of the embryonic gut. However, the mechanism by which the microbes from egg white are transferred to the embryonic gut is unknown. Water and proteins in egg white are transferred into the amniotic fluid for embryo consumption to support embryonic metabolism and growth, and this process also provides protection given the large amounts of antibacterial proteins in amniotic fluid [40, 46]. Thus, we hypothesize that the embryonic gut is colonized with microbes through the following process: microbes in egg white cross the amniotic membrane and then enter the amniotic fluid to colonize the embryonic gut after consumption by the embryo. We are interested in further studying this process in the future.

To comprehensively understand transcription in the embryonic small intestine, we performed RNA-seq and a series of bioinformatics analyses. The results showed that the top 30 enriched GO terms in the BP category for the significantly upregulated DEGs at E15 were associated with development, whereas those at E21 were associated with immunity (Fig. 5). The results of the IHC-P assays on intestinal mucosal immunity at E21 supported the GO enrichment results and showed that the levels of LYZ were increased in the duodenal, jejunal, and ileal mucosae, and that the level of IgA was increased in the jejunal mucosa (Fig. 6e). Although chick embryos do not secrete immunoglobulins [47], maternal immunoglobulins can be deposited in the intestinal mucosa to protect the embryo through various mechanisms, such as consumption and circulation [48]. In addition, intestinal epithelial cells can secrete LYZ beginning at E17 [49]. Regardless of whether they are maternally derived or synthesized by the embryo, IgA and LYZ are important factors for intestinal mucosal immunity and related maintenance of intestinal health [50, 51]. Thus, the results regarding the transcriptome and intestinal mucosal immunity explain and support the morphological results indicating that CCAB improves intestinal development during the embryonic period (Fig. 3). Our data suggest that the embryos arising from the CCAB group exhibited advanced intestinal development and intestinal immunity and were more fully prepared to face environmental challenges after hatching than those arising from the CON group.

The microbiota plays fundamental roles in the induction, education, and function of host immunity and in development [2]. Notably, in this study, the recognized probiotic *Bifidobacterium animalis* [52] was enriched in the magnum in the CCAB group, whereas taxa that may represent zoonotic pathogens (*Campylobacterales* [53] and *Helicobacteraceae* [54]) were enriched in the eCON group gut at E21 (Additional file 1: Fig. S7). We also noticed significantly different taxa between groups in each stage and hypothesized that these differences might influence embryonic intestinal development. We performed Spearman correlation analyses between upregulated DEGs and morphological indices and between upregulated DEGs and significantly enriched taxa (Fig. 6 a–d). The results showed that the expression of some of the DEGs was significantly positively correlated with the development of intestinal villi; in addition, the expression of some of the DEGs was highly correlated with the abundances of significantly enriched taxa that were also shared with magnum and egg white. These data indicated that the changes in intestinal morphology and the transcriptome in this study might be closely associated with the significantly enriched microbiota taxa in the embryonic gut. An increasing number of reports have confirmed that in mammals, maternal microbes are transferred to the infant gut during parturition, nursing, and contact, influencing the infant gut microbiota composition and driving immune development [55–57]. Maternal microbiota colonization has the ability to induce intestinal transcriptional reprogramming in offspring by targeting signature genes for cell division and differentiation and immune cells [58]. Maternal immune factors are able to ready neonatal innate immunity in time for the microbiota world, as well as the immune morphogenesis driven by the maternal microbiota [59, 60]. Therefore, the findings of this study suggest that maternal immune advantages shape and balance the microbiota in the reproductive system, and that resident microbes are vertically transferred into egg white for colonization of the offspring embryonic gut. The different maternal microbes that colonize the embryonic gut might be associated with the host intestinal transcriptome, adaptively driving intestinal development and immune establishment during the embryonic period. The transfer of reproductive system microbes from hens with enhanced maternal immunity might also provide advantages for offspring.

However, some limitations of this study should be noted. Data examined in this study were obtained from aggregated groups. The collection of paired mother–offspring samples was limited by the conflict between the multiple time-points of embryonic sampling and the reproduction physiology consisting of the laying cycle (one egg/hen/day), egg storage time (recommended 4–6 days), and egg hatchability (mortality and fertilization rate). The breeder hens were kept in cages, which also made egg tracking difficult. Data from paired mother–offspring could strengthen conclusions concerning maternal microbial transfer.

## Conclusion

Our data show that strong maternal immunity in birds benefits offspring intestinal immunity establishment and further contributes to intestinal development, thus ensuring offspring growth performance and health. Moreover, these adaptive maternal effects begin in the embryonic period. During this process, immune factors are transferred into eggs in a manner dependent on the maternal levels and provide adaptive protection for embryonic development. The maternal microbiota in the oviduct, which is shaped by the maternal immune system, is transferred to and possibly colonizes the embryonic gut to further drive general and immune-related intestinal development.

This study suggests that maternal immune advantages positively influence offspring health beginning in the embryonic period in birds. Our findings also indicate that the adaptive maternal effects might be realized via vertical transfer of high levels of maternal immune factors and via vertical transfer of the reproductive system microbiota shaped by strengthened maternal immunity. Given the results, we suggest that improved nutrition and health management to promote the immunity of breeder birds during the laying period may represent a win–win for the poultry farming industry chain: breeder bird farms, hatcheries, and rearing farms. The microbes in the maternal reproductive system may be useful resources for the promotion of animal health.

## Supplementary Information

**Additional file 1:** **Table S1.** Basal diet composition of experimental laying breeder hens and chicks. **Table S2.** Chick-brooding management. **Table S3.** Primers for qPCR. **Table S4.** Quality control of RNAs for sequencing. **Table S5.** Concentrations of purified PCR amplicons. **Table S6.** RNA-seq quality control of read filtration. **Table S7.** Mapping summary. **Table S8.** The 107 shared taxa among magnum, egg white, and E15 and E21 embryonic gut. **Figure S1.** Experimental design. **Figure S2.** Electrophoresis results of PCR amplification products with positive and negative controls. Marker = 2000, 1000, 750, 500, 250 and 100 bp from top to bottom (TaKaRa, DL2000 DNA Marker). **Figure S3.** Gene coverage of each sample for mapping quality control. CON and CCAB are abbreviated as A and C in the figure. **Figure S4.** (a). Growth performance of the offspring chicks from 1 to 21 d of age. Data are shown as the means + SEMs. Student’s t test was conducted. ns *P* ≥ 0.05, *P < 0.05 and **P < 0.01. n = 30 chicks. (b) Embryo mortality in the CON (left) and CCAB (right) groups. Embryo mortality = dead embryo number/fertile egg number. Set egg number = 900 eggs in each group. The number of fertile eggs was 792 and 797 in the CON and CCAB groups, respectively. **Figure S5.** IgA, IgG, IgM, LYZ and AvBDs levels in embryonic serum (a) and egg white (b); the levels of IgA, IgG and IgM in embryonic yolk sac fluid (c); and the levels of LYZ and AvBDs in amniotic fluid (d) as determined by ELISA. Data are means ± SEMs. Student’s t test was conducted. ns *P* ≥ 0.05 and *P < 0.05. Figure S6. Microbial composition at the phylum (a) and genus (b) levels in the magnum (abbreviated as mag), egg white (abbreviated as EW), and E15 and E21 embryonic gut in each sample. Figure S7. Significantly enriched taxa (log10(LDA score) > 2, *P* < 0.05) in the magnum (a; abbreviated as mag), egg white (b; abbreviated as EW), and embryonic gut at E15 (c) and E21 (d) between groups as determined by LEfSe with Kruskal‒Wallis and Wilcoxon tests and the one against-all strategy.

**Additional file 2:** Sheet 1: Significantly upregulated DEGs in the small intestine at E15 in the eCCAB group compared with the eCON group. Sheet 2: Significantly upregulated DEGs in the small intestine at E21 in the eCCAB group compared with the eCON group. Sheet 3: Significantly enriched GO terms in the BP category for the upregulated DEGs in the small intestine at E15 in the eCCAB group compared with the eCON group. Sheet 4: Significantly enriched GO terms in the BP category for the upregulated DEGs in the small intestine at E21 in the eCCAB group compared with the eCON group.

## Data Availability

The raw sequences from 16S rRNA sequencing and RNA-seq for this study are available at the National Center for Biotechnology Information (NCBI) Sequence Read Archive (SRA) under the BioProject accession numbers PRJNA694626 and PRJNA694787 (https://www.ncbi.nlm.nih.gov/).

## Abbreviations

CON, eCON, and cCON: Laying breeder hen, offspring embryo, and offspring chick control group, respectively
CCAB, eCCAB, and cCCAB: Laying breeder hen, offspring embryo, and offspring chick experimental group, respectively
E: Embryonic day
RNA-seq: RNA sequencing
IHC-P: Immunohistochemistry paraffin
FPKM: Fragments per kilobase per million mapped reads
PCA: Principal component analysis
DEGs: Differentially expressed genes
GO: Gene Ontology
ASVs: Amplicon sequence variants
NMDS: Nonmetric multidimensional scaling
LEfSe: Linear discriminant analysis effect size
LYZ: Lysozyme
AvBDs: Avian beta-defensins
BP: Biological process
