# Supplementary material for: Maternal effects drive intestinal development beginning in the embryonic period on the basis of maternal immune and microbial transfer in chickens
Source: Microbiome. 2023 Mar 3;11:41. doi: 10.1186/s40168-023-01490-5 (PMC9983169; doi:10.1186/s40168-023-01490-5)
Supplement: Supplementary file 2 — Additional file 1: Table S1. Basal diet composition of experimental laying breeder hens and chicks. Table S2. Chick-brooding management. Table S3. Primers for qPCR. Table S4. Quality control of RNAs for sequencing. Table S5. Concentrations of purified PCR amplicons. Table S6. RNA-seq quality control of read filtration. Table S7. Mapping summary. Table S8. The 107 shared taxa among magnum, egg white, and E15 and E21 embryonic gut. Figure S1. Experimental design. Figure S2. Electrophoresis results of PCR amplification products with positive and negative controls. Marker = 2000, 1000, 750, 500, 250 and 100 bp from top to bottom (TaKaRa, DL2000 DNA Marker). Figure S3. Gene coverage of each sample for mapping quality control. CON and CCAB are abbreviated as A and C in the figure. Figure S4. (a). Growth performance of the offspring chicks from 1 to 21 d of age. Data are shown as the means + SEMs. Student’s t test was conducted. ns P ≥ 0.05, *P < 0.05 and **P < 0.01. n = 30 chicks. (b) Embryo mortality in the CON (left) and CCAB (right) groups. Embryo mortality = dead embryo number/fertile egg number. Set egg number = 900 eggs in each group. The number of fertile eggs was 792 and 797 in the CON and CCAB groups, respectively. Figure S5. IgA, IgG, IgM, LYZ and AvBDs levels in embryonic serum (a) and egg white (b); the levels of IgA, IgG and IgM in embryonic yolk sac fluid (c); and the levels of LYZ and AvBDs in amniotic fluid (d) as determined by ELISA. Data are means ± SEMs. Student’s t test was conducted. ns P ≥ 0.05 and *P < 0.05. Figure S6. Microbial composition at the phylum (a) and genus (b) levels in the magnum (abbreviated as mag), egg white (abbreviated as EW), and E15 and E21 embryonic gut in each sample. Figure S7. Significantly enriched taxa (log10(LDA score) > 2, P < 0.05) in the magnum (a; abbreviated as mag), egg white (b; abbreviated as EW), and embryonic gut at E15 (c) and E21 (d) between groups as determined by LEfSe with Kruskal‒Wallis and Wilcoxon tests a [file 40168_2023_1490_MOESM1_ESM.docx]

**Table S1.** Basal diet composition of experimental laying breeder hens and chicks.

| Items | Laying breeder hen diet | Chick diet |
| --- | --- | --- |
| Ingredient (%) |  |  |
| Corn | 60.00 | 56.19 |
| Soybean meal | 22.30 | 19.61 |
| Limestone | 9.00 | 1.53 |
| Wheat middling and red dog | 3.00 | 8.00 |
| Soybean oil | 1.50 | - |
| Puffed soybean | - | 8.00 |
| Corn gluten meal | - | 2.00 |
| Fish meal | - | 2.00 |
| CaHPO_4_ | 1.50 | 1.10 |
| Premix^a^ | 1.40 | 1.00 |
| Choline chloride | 0.45 | 0.06 |
| NaCl | - | 0.25 |
| Lys | 0.35 | 0.15 |
| Thr | 0.30 | 0.10 |
| Met | 0.20 | 0.01 |
| Total | 100 | 100 |
|  |  |  |
| Calculated composition^b^ (%) | |  |
| ME(KJ/kg) | 11.22 | 12.90 |
| CP | 15.85 | 20.20 |
| Ca | 3.76 | 1.05 |
| Available phosphorus | 0.460 | 0.380 |
| Lys | 1.100 | 1.128 |
| Methionine | 0.430 | 0.487 |
| Met+Cys | 0.726 | 0.847 |

^a^The premix provided the following per kg of diet: Vitamin A 8000 IU, Vitamin D 3750 IU, Vitamin E 100 mg, Vitamin K3 3 mg, Vitamin B2 12.5 mg, Vitamin B6 9 mg, Vitamin B12 0.03 mg, pantothenic acid 18 mg, niacin 60 mg, folic acid 1.5 mg, biotin 0.225 mg, Fe 80 mg, Cu 9 mg, I 0.9 mg, Se 0.3 mg, Mn 12.55 mg, and Zn 25.2 mg.

^b^Values were calculated from data provide by the China Feed Database (2013).

**Table S2.** Chick-brooding management.

| Age | 0-3 D | 4-7 D | 8-14 D | 15-21 D |
| --- | --- | --- | --- | --- |
| Air temperature (℃) | 35-36 | 33-35 | 31-33 | 29-31 |
| Light intensity (Lux) | 30-50 | 30-50 | 25 | 25 |
| Light hours (h) | 22 | 21 | 20 | 19 |
| Relative humidity (%) | 60 | 60 | 60-40 | 60-40 |

**Table S3.** Primers for qPCR.

| Gene | Primer sequences (5'-3') | Identifier |
| --- | --- | --- |
| *AvBD10* | F: GCTCAGCAGACCCACTTTTC | NM_001001609.1 |
|  | R: GTTGCTGGTACAAGGGCAAT |  |
| *AvBD11* | F: ACTGCATCCGTTCCAAAGTC | NM_001001779.1 |
|  | R: TGTGGCTTTCTGCAATTCTG |  |
| *AvBD12* | F: GGGGATTGTGCCGAGTGGGG | NM_001001607.2 |
|  | R: TGCTGGAGGTGCTGCTGCTC |  |
| *AvBD13* | F: GCCATCGTTGTCATTCTCCTCCTC | NM_001001780.1 |
|  | R: CTCCCAGCCCAGCTCTCCATG |  |
| *LYZ* | F: GGGAAACTGGGTGTGTGTTGCA | FJ542564.1 |
|  | R: TCTTCTTCGCGCAGTTCACGCT |  |
| *Avidin* | F: CTGCATGGGACACAAAACAC | NM_205320.1 |
|  | R: TTAACACTTGACCGCAGCAG |  |
| *Gallin* | F: CTCCAGCCTCGCTCACAC | FN550409.1 |
|  | R: TTGAGAGGAGGGGATGACAC |  |
| *Ovoinhibitor* | F: TAAGGATGGCAGGACTTTGG | NM_001030612.1 |
|  | R: GAGTTTGCCACCAGTGGTTT |  |
| *Ovomucoid* | F: TGCAGTCGTGGAAAGCAACGG | FJ227543.1 |
|  | R: GCTGAGCTCCCCAGAGTGCGA |  |
| *Cystatin* | F: ACAACTTGCCCCAAGTCATC | NM_205500.2 |
|  | R: GGCAGCGATACAATCCATCT |  |
| *TLR4* | F: GCCATCCCAACCCAACCACAG | NM_001030693.1 |
|  | R: CCACTGAGCAGCACCAATGAGTAG |  |
| *FcRL4* | F: CAGGAGGGACAGGTGATGGAAGG | NM_001097529.2 |
|  | R: TCGGTGCCAGGAGAAGGAGATG |  |
| *β-actin* | F: GCCCTGGCACCTAGCACAATG | NM205518.1 |
|  | R: CTCCTGCTTGCTGATCCACATCTG |  |

**Table S4.** Quality control of RNAs for sequencing.

| Sample ID | OD260/280 | OD260/230 | RIN | 28S/18S |
| --- | --- | --- | --- | --- |
| E15eCON1 | 2.02 | 2.127 | 8.5 | 1.3 |
| E15eCON2 | 2.02 | 2.259 | 9 | 1.5 |
| E15eCON3 | 2.017 | 2.274 | 9.9 | 1.5 |
| E15eCON4 | 2.075 | 2.141 | 9.4 | 1.8 |
| E15eCON5 | 2.04 | 2.193 | 9.7 | 1.7 |
| E15eCON6 | 2.045 | 2.222 | 9.8 | 1.6 |
| E15eCCAB1 | 2.039 | 2.219 | 9.7 | 1.7 |
| E15eCCAB2 | 2.046 | 2.227 | 9.3 | 1.6 |
| E15eCCAB3 | 2.035 | 2.081 | 9.6 | 1.7 |
| E15eCCAB4 | 2.041 | 1.686 | 9.6 | 1.7 |
| E15eCCAB5 | 2.02 | 2.226 | 9.6 | 1.6 |
| E15eCCAB6 | 2.023 | 1.887 | 9.7 | 1.6 |
| E21eCON1 | 1.992 | 2.27 | 8.8 | 1.7 |
| E21eCON2 | 2.059 | 2.232 | 9.3 | 1.5 |
| E21eCON3 | 2.006 | 2.288 | 9.1 | 1.7 |
| E21eCON4 | 2.053 | 2.19 | 9.3 | 1.7 |
| E21eCON5 | 2.089 | 2.214 | 8.7 | 1.5 |
| E21eCON6 | 2.083 | 2.24 | 8.7 | 2.2 |
| E21eCCAB1 | 2.081 | 2.199 | 9 | 1.7 |
| E21eCCAB2 | 2.079 | 2.23 | 9 | 1.8 |
| E21eCCAB3 | 2.094 | 2.189 | 9.4 | 1.7 |
| E21eCCAB4 | 2.051 | 2.21 | 9.4 | 1.7 |
| E21eCCAB5 | 2.064 | 2.222 | 9.1 | 1.7 |
| E21eCCAB6 | 2.072 | 2.208 | 9.4 | 1.5 |

**Table S5.** Concentrations of purified PCR amplicons.

| Sample ID | Length (bp) | Concentration (ng/μl) | | Sample ID | Length (bp) | Concentration (ng/μl) |
| --- | --- | --- | --- | --- | --- | --- |
| CON_mag1 | 500bp | 13.71 |  | E15eCON1 | 500bp | 6.63 |
| CON_mag2 | 500bp | 6.66 |  | E15eCON2 | 500bp | 6.44 |
| CON_mag3 | 500bp | 18.22 |  | E15eCON3 | 500bp | 7.8 |
| CON_mag4 | 500bp | 9.25 |  | E15eCON4 | 500bp | 8.78 |
| CON_mag5 | 500bp | 12.9 |  | E15eCON5 | 500bp | 7.58 |
| CON_mag6 | 500bp | 9.49 |  | E15eCON6 | 500bp | 7.43 |
| CCAB_mag1 | 500bp | 8.89 |  | E15eCCAB1 | 500bp | 8.6 |
| CCAB_mag2 | 500bp | 5.32 |  | E15eCCAB2 | 500bp | 5.64 |
| CCAB_mag3 | 500bp | 8.59 |  | E15eCCAB3 | 500bp | 6.48 |
| CCAB_mag4 | 500bp | 11.78 |  | E15eCCAB4 | 500bp | 8.25 |
| CCAB_mag5 | 500bp | 14.59 |  | E15eCCAB5 | 500bp | 6.15 |
| CCAB_mag6 | 500bp | 11.25 |  | E15eCCAB6 | 500bp | 7.52 |
| CON_EW1 | 500bp | 0.65 |  | E21eCON1 | 500bp | 1.66 |
| CON_EW2 | 500bp | 2.26 |  | E21eCON2 | 500bp | 0.86 |
| CON_EW3 | 500bp | 3.39 |  | E21eCON3 | 500bp | 2.04 |
| CON_EW4 | 500bp | 3.23 |  | E21eCON4 | 500bp | 0.55 |
| CON_EW5 | 500bp | 5.48 |  | E21eCON5 | 500bp | 3.54 |
| CON_EW6 | 500bp | 6.88 |  | E21eCON6 | 500bp | 5.02 |
| CCAB_EW1 | 500bp | 2.44 |  | E21eCCAB1 | 500bp | 5.46 |
| CCAB_EW2 | 500bp | 3.14 |  | E21eCCAB2 | 500bp | 7.06 |
| CCAB_EW3 | 500bp | 9.75 |  | E21eCCAB3 | 500bp | 5.11 |
| CCAB_EW4 | 500bp | 8.56 |  | E21eCCAB4 | 500bp | 11.58 |
| CCAB_EW5 | 500bp | 11.07 |  | E21eCCAB5 | 500bp | 6.7 |
| CCAB_EW6 | 500bp | 8.94 |  | E21eCCAB6 | 500bp | 9.72 |

**Table S6.** RNA-seq quality control of read filtration.

| Sample ID | Clean Read Number | Clean Bases | Clean Read% | Clean Bases% |
| --- | --- | --- | --- | --- |
| E15eCON1 | 41558148 | 6233722200 | 93.33 | 93.33 |
| E15eCON2 | 41360386 | 6204057900 | 93.28 | 93.28 |
| E15eCON3 | 42418230 | 6362734500 | 93.33 | 93.33 |
| E15eCON4 | 40644662 | 6096699300 | 93.62 | 93.62 |
| E15eCON5 | 41386504 | 6207975600 | 93.12 | 93.12 |
| E15eCON6 | 38951086 | 5842662900 | 93.68 | 93.68 |
| E15eCCAB1 | 41662950 | 6249442500 | 93.74 | 93.74 |
| E15eCCAB2 | 42688444 | 6403266600 | 93.56 | 93.56 |
| E15eCCAB3 | 42317908 | 6347686200 | 93.53 | 93.53 |
| E15eCCAB4 | 47601160 | 7140174000 | 94.09 | 94.09 |
| E15eCCAB5 | 44086722 | 6613008300 | 93.55 | 93.55 |
| E15eCCAB6 | 44799120 | 6719868000 | 93.42 | 93.42 |
| E21eCON1 | 40306876 | 6046031400 | 93.72 | 93.72 |
| E21eCON2 | 44271440 | 6640716000 | 93.68 | 93.68 |
| E21eCON3 | 42630124 | 6394518600 | 93.88 | 93.88 |
| E21eCON4 | 44743648 | 6711547200 | 92.61 | 92.61 |
| E21eCON5 | 44383120 | 6657468000 | 93.62 | 93.62 |
| E21eCON6 | 40798558 | 6119783700 | 93.37 | 93.37 |
| E21eCCAB1 | 48437538 | 7265630700 | 93.67 | 93.67 |
| E21eCCAB2 | 48034996 | 7205249400 | 93.73 | 93.73 |
| E21eCCAB3 | 41246722 | 6187008300 | 93.92 | 93.92 |
| E21eCCAB4 | 43833514 | 6575027100 | 93.68 | 93.68 |
| E21eCCAB5 | 48884344 | 7332651600 | 93.6 | 93.6 |
| E21eCCAB6 | 48159636 | 7223945400 | 93.29 | 93.29 |

**Table S7.** Mapping summary.

| Sample ID | Clean Reads | Total Mapped | Multiple Mapped | Uniquely Mapped | Map Events | Mapped to Gene | Mapped to InterGene | Mapped to Exon |
| --- | --- | --- | --- | --- | --- | --- | --- | --- |
| E15eCON1 | 41558148 | 37974791 (91.38%) | 1483471 (3.91%) | 36491320 (96.09%) | 36491320 | 30208501 (82.78%) | 6282819 (17.22%) | 24501986 (81.11%) |
| E15eCON2 | 41360386 | 37568364 (90.83%) | 962838 (2.56%) | 36605526 (97.44%) | 36605526 | 30567812 (83.51%) | 6037714 (16.49%) | 26497850 (86.69%) |
| E15eCON3 | 42418230 | 38264770 (90.21%) | 834196 (2.18%) | 37430574 (97.82%) | 37430574 | 31185022 (83.31%) | 6245552 (16.69%) | 26791400 (85.91%) |
| E15eCON4 | 40644662 | 36937863 (90.88%) | 1122406 (3.04%) | 35815457 (96.96%) | 35815457 | 29991932 (83.74%) | 5823525 (16.26%) | 26105396 (87.04%) |
| E15eCON5 | 41386504 | 38049564 (91.94%) | 808507 (2.12%) | 37241057 (97.88%) | 37241057 | 31263698 (83.95%) | 5977359 (16.05%) | 26965302 (86.25%) |
| E15eCON6 | 38951086 | 35755482 (91.80%) | 800441 (2.24%) | 34955041 (97.76%) | 34955041 | 29445004 (84.24%) | 5510037 (15.76%) | 25677920 (87.21%) |
| E15eCCAB1 | 41662950 | 38327754 (91.99%) | 860979 (2.25%) | 37466775 (97.75%) | 37466775 | 31433469 (83.90%) | 6033306 (16.10%) | 27151631 (86.38%) |
| E15eCCAB2 | 42688444 | 39032903 (91.44%) | 917207 (2.35%) | 38115696 (97.65%) | 38115696 | 31956489 (83.84%) | 6159207 (16.16%) | 27973560 (87.54%) |
| E15eCCAB3 | 42317908 | 38970844 (92.09%) | 904869 (2.32%) | 38065975 (97.68%) | 38065975 | 32102035 (84.33%) | 5963940 (15.67%) | 27821667 (86.67%) |
| E15eCCAB4 | 47601160 | 44001787 (92.44%) | 1112197 (2.53%) | 42889590 (97.47%) | 42889590 | 36095386 (84.16%) | 6794204 (15.84%) | 30896930 (85.60%) |
| E15eCCAB5 | 44086722 | 40531481 (91.94%) | 974455 (2.40%) | 39557026 (97.60%) | 39557026 | 33327543 (84.25%) | 6229483 (15.75%) | 29102718 (87.32%) |
| E15eCCAB6 | 44799120 | 41362740 (92.33%) | 1046979 (2.53%) | 40315761 (97.47%) | 40315761 | 34021364 (84.39%) | 6294397 (15.61%) | 29331181 (86.21%) |
| E21eCON1 | 40306876 | 36844562 (91.41%) | 916017 (2.49%) | 35928545 (97.51%) | 35928545 | 30505896 (84.91%) | 5422649 (15.09%) | 26770053 (87.75%) |
| E21eCON2 | 44271440 | 40414740 (91.29%) | 969640 (2.40%) | 39445100 (97.60%) | 39445100 | 33421184 (84.73%) | 6023916 (15.27%) | 29254376 (87.53%) |
| E21eCON3 | 42630124 | 38775581 (90.96%) | 931386 (2.40%) | 37844195 (97.60%) | 37844195 | 31991420 (84.53%) | 5852775 (15.47%) | 28325784 (88.54%) |
| E21eCON4 | 44743648 | 40383270 (90.25%) | 965757 (2.39%) | 39417513 (97.61%) | 39417513 | 33467783 (84.91%) | 5949730 (15.09%) | 29275760 (87.47%) |
| E21eCON5 | 44383120 | 40540397 (91.34%) | 1038680 (2.56%) | 39501717 (97.44%) | 39501717 | 33982228 (86.03%) | 5519489 (13.97%) | 30535873 (89.86%) |
| E21eCON6 | 40798558 | 37217668 (91.22%) | 908989 (2.44%) | 36308679 (97.56%) | 36308679 | 31088474 (85.62%) | 5220205 (14.38%) | 27974919 (89.98%) |
| E21eCCAB1 | 48437538 | 44130621 (91.11%) | 1126206 (2.55%) | 43004415 (97.45%) | 43004415 | 36568438 (85.03%) | 6435977 (14.97%) | 31989617 (87.48%) |
| E21eCCAB2 | 48034996 | 43993601 (91.59%) | 1068341 (2.43%) | 42925260 (97.57%) | 42925260 | 36742159 (85.60%) | 6183101 (14.40%) | 32610677 (88.76%) |
| E21eCCAB3 | 41246722 | 37610992 (91.19%) | 893740 (2.38%) | 36717252 (97.62%) | 36717252 | 31552449 (85.93%) | 5164803 (14.07%) | 28255364 (89.55%) |
| E21eCCAB4 | 43833514 | 40113551 (91.51%) | 901707 (2.25%) | 39211844 (97.75%) | 39211844 | 33727518 (86.01%) | 5484326 (13.99%) | 29982724 (88.90%) |
| E21eCCAB5 | 48884344 | 44734595 (91.51%) | 1117116 (2.50%) | 43617479 (97.50%) | 43617479 | 37223107 (85.34%) | 6394372 (14.66%) | 32960223 (88.55%) |
| E21eCCAB6 | 48159636 | 44027990 (91.42%) | 1062790 (2.41%) | 42965200 (97.59%) | 42965200 | 36790044 (85.63%) | 6175156 (14.37%) | 32171248 (87.45%) |

**Table S8.** The 107 shared taxa among magnum, egg white, and E15 and E21 embryonic gut.

| ASV ID | Taxonomy |
| --- | --- |
| ASV_8454 | *d__Bacteria; p__Proteobacteria; c__Betaproteobacteria; o__Burkholderiales; f__Comamonadaceae; g__unclassified_Comamonadaceae; s__unclassified_Comamonadaceae* |
| ASV_70489 | *d__Bacteria; p__Firmicutes; c__Bacilli; o__Lactobacillales; f__Lactobacillaceae; g__Lactobacillus; s__unidentified_Lactobacillus* |
| ASV_42676 | *d__Bacteria; p__Proteobacteria; c__Gammaproteobacteria; o__Pseudomonadales; f__Moraxellaceae; g__Acinetobacter; s__Acinetobacter_johnsonii* |
| ASV_33289 | *d__Bacteria; p__Proteobacteria; c__Betaproteobacteria; o__unidentified_Betaproteobacteria; f__unidentified_Betaproteobacteria; g__unidentified_Betaproteobacteria; s__unidentified_Betaproteobacteria* |
| ASV_71002 | *d__Bacteria; p__Proteobacteria; c__Betaproteobacteria; o__Burkholderiales; f__Comamonadaceae; g__unclassified_Comamonadaceae; s__unclassified_Comamonadaceae* |
| ASV_66400 | *d__Bacteria; p__Proteobacteria; c__Betaproteobacteria; o__Burkholderiales; f__Comamonadaceae; g__unidentified_Comamonadaceae; s__unidentified_Comamonadaceae* |
| ASV_31767 | *d__Bacteria; p__Proteobacteria; c__Betaproteobacteria; o__Burkholderiales; f__Comamonadaceae; g__Roseateles; s__unclassified_Roseateles* |
| ASV_41113 | *d__Bacteria; p__Firmicutes; c__Clostridia; o__Clostridiales; f__Lachnospiraceae; g__unclassified_Lachnospiraceae; s__unclassified_Lachnospiraceae* |
| ASV_64952 | *d__Bacteria; p__Proteobacteria; c__Alphaproteobacteria; o__Rhizobiales; f__Hyphomicrobiaceae; g__unclassified_Hyphomicrobiaceae; s__unclassified_Hyphomicrobiaceae* |
| ASV_17409 | *d__Bacteria; p__Proteobacteria; c__Betaproteobacteria; o__Rhodocyclales; f__Rhodocyclaceae; g__KD1-23; s__unidentified_KD1-23* |
| ASV_41193 | *d__Bacteria; p__Bacteroidetes; c__Bacteroidia; o__Bacteroidales; f__S24-7; g__unidentified_S24-7; s__unidentified_S24-7* |
| ASV_34807 | *d__Bacteria; p__Proteobacteria; c__Betaproteobacteria; o__Burkholderiales; f__Comamonadaceae; g__unclassified_Comamonadaceae; s__unclassified_Comamonadaceae* |
| ASV_55310 | *d__Bacteria; p__Proteobacteria; c__Epsilonproteobacteria; o__Campylobacterales; f__Helicobacteraceae; g__Helicobacter; s__unclassified_Helicobacter* |
| ASV_63489 | *d__Bacteria; p__Bacteroidetes; c__Bacteroidia; o__Bacteroidales; f__S24-7; g__unidentified_S24-7; s__unidentified_S24-7* |
| ASV_65472 | *d__Bacteria; p__Proteobacteria; c__Betaproteobacteria; o__Burkholderiales; f__Comamonadaceae; g__Rubrivivax; s__unidentified_Rubrivivax* |
| ASV_2192 | *d__Bacteria; p__Proteobacteria; c__Betaproteobacteria; o__Burkholderiales; f__Comamonadaceae; g__Methylibium; s__unclassified_Methylibium* |
| ASV_28599 | *d__Bacteria; p__Bacteroidetes; c__Bacteroidia; o__Bacteroidales; f__Prevotellaceae; g__Prevotella; s__unidentified_Prevotella* |
| ASV_59304 | *d__Bacteria; p__Firmicutes; c__Bacilli; o__Bacillales; f__Staphylococcaceae; g__Staphylococcus; s__Staphylococcus_sciuri* |
| ASV_61808 | *d__Bacteria; p__Proteobacteria; c__Betaproteobacteria; o__Burkholderiales; f__Comamonadaceae; g__unclassified_Comamonadaceae; s__unclassified_Comamonadaceae* |
| ASV_35993 | *d__Bacteria; p__Proteobacteria; c__Betaproteobacteria; o__Burkholderiales; f__Comamonadaceae; g__unclassified_Comamonadaceae; s__unclassified_Comamonadaceae* |
| ASV_37777 | *d__Bacteria; p__Bacteroidetes; c__Bacteroidia; o__Bacteroidales; f__S24-7; g__unidentified_S24-7; s__unidentified_S24-7* |
| ASV_50391 | *d__Bacteria; p__Proteobacteria; c__Betaproteobacteria; o__Burkholderiales; f__Comamonadaceae; g__unclassified_Comamonadaceae; s__unclassified_Comamonadaceae* |
| ASV_1700 | *d__Bacteria; p__Bacteroidetes; c__Bacteroidia; o__Bacteroidales; f__S24-7; g__unidentified_S24-7; s__unidentified_S24-7* |
| ASV_33766 | *d__Bacteria; p__Firmicutes; c__Bacilli; o__Lactobacillales; f__Lactobacillaceae; g__Lactobacillus; s__Lactobacillus_hamsteri* |
| ASV_54509 | *d__Bacteria; p__Proteobacteria; c__Betaproteobacteria; o__Burkholderiales; f__Comamonadaceae; g__unclassified_Comamonadaceae; s__unclassified_Comamonadaceae* |
| ASV_55431 | *d__Bacteria; p__Proteobacteria; c__Betaproteobacteria; o__Burkholderiales; f__Alcaligenaceae; g__Sutterella; s__unidentified_Sutterella* |
| ASV_15115 | *d__Bacteria; p__Bacteroidetes; c__Bacteroidia; o__Bacteroidales; f__S24-7; g__unidentified_S24-7; s__unidentified_S24-7* |
| ASV_34631 | *d__Bacteria; p__Bacteroidetes; c__Bacteroidia; o__Bacteroidales; f__S24-7; g__unidentified_S24-7; s__unidentified_S24-7* |
| ASV_2248 | *d__Bacteria; p__Firmicutes; c__Bacilli; o__Lactobacillales; f__Lactobacillaceae; g__Lactobacillus; s__unidentified_Lactobacillus* |
| ASV_26498 | *d__Bacteria; p__Bacteroidetes; c__Bacteroidia; o__Bacteroidales; f__S24-7; g__unidentified_S24-7; s__unidentified_S24-7* |
| ASV_66889 | *d__Bacteria; p__Bacteroidetes; c__Bacteroidia; o__Bacteroidales; f__S24-7; g__unidentified_S24-7; s__unidentified_S24-7* |
| ASV_58313 | *d__Bacteria; p__Firmicutes; c__Bacilli; o__Lactobacillales; f__Lactobacillaceae; g__Lactobacillus; s__Lactobacillus_vaginalis* |
| ASV_34294 | *d__Bacteria; p__Proteobacteria; c__Betaproteobacteria; o__Rhodocyclales; f__Rhodocyclaceae; g__KD1-23; s__unidentified_KD1-23* |
| ASV_56476 | *d__Bacteria; p__Proteobacteria; c__Gammaproteobacteria; o__Pseudomonadales; f__Moraxellaceae; g__Acinetobacter; s__unclassified_Acinetobacter* |
| ASV_1343 | *d__Bacteria; p__Proteobacteria; c__Gammaproteobacteria; o__Enterobacteriales; f__Enterobacteriaceae; g__Shigella; s__unclassified_Shigella* |
| ASV_6627 | *d__Bacteria; p__Proteobacteria; c__Betaproteobacteria; o__Burkholderiales; f__Comamonadaceae; g__unclassified_Comamonadaceae; s__unclassified_Comamonadaceae* |
| ASV_36098 | *d__Bacteria; p__Proteobacteria; c__Betaproteobacteria; o__Burkholderiales; f__Comamonadaceae; g__unclassified_Comamonadaceae; s__unclassified_Comamonadaceae* |
| ASV_20092 | *d__Bacteria; p__Proteobacteria; c__Gammaproteobacteria; o__Enterobacteriales; f__Enterobacteriaceae; g__unclassified_Enterobacteriaceae; s__unclassified_Enterobacteriaceae* |
| ASV_29232 | *d__Bacteria; p__Bacteroidetes; c__Bacteroidia; o__Bacteroidales; f__S24-7; g__unidentified_S24-7; s__unidentified_S24-7* |
| ASV_34602 | *d__Bacteria; p__Proteobacteria; c__Betaproteobacteria; o__Burkholderiales; f__unclassified_Burkholderiales; g__unclassified_Burkholderiales; s__unclassified_Burkholderiales* |
| ASV_47536 | *d__Bacteria; p__Proteobacteria; c__Gammaproteobacteria; o__Pseudomonadales; f__Moraxellaceae; g__Acinetobacter; s__Acinetobacter_rhizosphaerae* |
| ASV_49870 | *d__Bacteria; p__Firmicutes; c__Clostridia; o__Clostridiales; f__Ruminococcaceae; g__Faecalibacterium; s__Faecalibacterium_prausnitzii* |
| ASV_43183 | *d__Bacteria; p__Firmicutes; c__Clostridia; o__Clostridiales; f__Ruminococcaceae; g__Faecalibacterium; s__Faecalibacterium_prausnitzii* |
| ASV_44716 | *d__Bacteria; p__Firmicutes; c__Bacilli; o__Bacillales; f__Staphylococcaceae; g__Staphylococcus; s__unclassified_Staphylococcus* |
| ASV_2742 | *d__Bacteria; p__Firmicutes; c__Clostridia; o__Clostridiales; f__Ruminococcaceae; g__Oscillospira; s__unidentified_Oscillospira* |
| ASV_34793 | *d__Bacteria; p__Proteobacteria; c__Betaproteobacteria; o__Burkholderiales; f__Comamonadaceae; g__unclassified_Comamonadaceae; s__unclassified_Comamonadaceae* |
| ASV_34171 | *d__Bacteria; p__Proteobacteria; c__Alphaproteobacteria; o__Rhizobiales; f__Hyphomicrobiaceae; g__Rhodoplanes; s__Rhodoplanes_elegans* |
| ASV_46800 | *d__Bacteria; p__Proteobacteria; c__Betaproteobacteria; o__Burkholderiales; f__Comamonadaceae; g__unclassified_Comamonadaceae; s__unclassified_Comamonadaceae* |
| ASV_64968 | *d__Bacteria; p__Proteobacteria; c__Gammaproteobacteria; o__Pseudomonadales; f__Moraxellaceae; g__unclassified_Moraxellaceae; s__unclassified_Moraxellaceae* |
| ASV_46560 | *d__Bacteria; p__Firmicutes; c__Clostridia; o__Clostridiales; f__Ruminococcaceae; g__Ruminococcus; s__unidentified_Ruminococcus* |
| ASV_21597 | *d__Bacteria; p__Proteobacteria; c__Gammaproteobacteria; o__Enterobacteriales; f__Enterobacteriaceae; g__Shigella; s__unclassified_Shigella* |
| ASV_5528 | *d__Bacteria; p__Proteobacteria; c__Betaproteobacteria; o__Burkholderiales; f__unidentified_Burkholderiales; g__unidentified_Burkholderiales; s__unidentified_Burkholderiales* |
| ASV_49520 | *d__Bacteria; p__Proteobacteria; c__Betaproteobacteria; o__Burkholderiales; f__Comamonadaceae; g__unclassified_Comamonadaceae; s__unclassified_Comamonadaceae* |
| ASV_5238 | *d__Bacteria; p__Firmicutes; c__Clostridia; o__Clostridiales; f__Ruminococcaceae; g__Oscillospira; s__unidentified_Oscillospira* |
| ASV_7608 | *d__Bacteria; p__Proteobacteria; c__Betaproteobacteria; o__Neisseriales; f__Neisseriaceae; g__Neisseria; s__unclassified_Neisseria* |
| ASV_16533 | *d__Bacteria; p__Proteobacteria; c__Betaproteobacteria; o__Burkholderiales; f__Comamonadaceae; g__unclassified_Comamonadaceae; s__unclassified_Comamonadaceae* |
| ASV_69226 | *d__Bacteria; p__Proteobacteria; c__Gammaproteobacteria; o__Pseudomonadales; f__Moraxellaceae; g__Acinetobacter; s__Acinetobacter_johnsonii* |
| ASV_11311 | *d__Bacteria; p__Proteobacteria; c__Gammaproteobacteria; o__Pseudomonadales; f__Moraxellaceae; g__Acinetobacter; s__Acinetobacter_lwoffii* |
| ASV_12732 | *d__Bacteria; p__Proteobacteria; c__Betaproteobacteria; o__Burkholderiales; f__Comamonadaceae; g__unclassified_Comamonadaceae; s__unclassified_Comamonadaceae* |
| ASV_61638 | *d__Bacteria; p__Bacteroidetes; c__Bacteroidia; o__Bacteroidales; f__S24-7; g__unidentified_S24-7; s__unidentified_S24-7* |
| ASV_7717 | *d__Bacteria; p__Proteobacteria; c__Alphaproteobacteria; o__Caulobacterales; f__Caulobacteraceae; g__Caulobacter; s__unidentified_Caulobacter* |
| ASV_48239 | *d__Bacteria; p__Proteobacteria; c__Deltaproteobacteria; o__Desulfovibrionales; f__Desulfovibrionaceae; g__unidentified_Desulfovibrionaceae; s__unidentified_Desulfovibrionaceae* |
| ASV_41388 | *d__Bacteria; p__Proteobacteria; c__Betaproteobacteria; o__Burkholderiales; f__Comamonadaceae; g__unclassified_Comamonadaceae; s__unclassified_Comamonadaceae* |
| ASV_6597 | *d__Bacteria; p__Bacteroidetes; c__Bacteroidia; o__Bacteroidales; f__S24-7; g__unidentified_S24-7; s__unidentified_S24-7* |
| ASV_15694 | *d__Bacteria; p__Bacteroidetes; c__Bacteroidia; o__Bacteroidales; f__[Odoribacteraceae]; g__Odoribacter; s__unidentified_Odoribacter* |
| ASV_59306 | *d__Bacteria; p__Proteobacteria; c__Gammaproteobacteria; o__Enterobacteriales; f__Enterobacteriaceae; g__Shigella; s__unclassified_Shigella* |
| ASV_35963 | *d__Bacteria; p__Proteobacteria; c__Betaproteobacteria; o__Burkholderiales; f__Comamonadaceae; g__unclassified_Comamonadaceae; s__unclassified_Comamonadaceae* |
| ASV_18923 | *d__Bacteria; p__Bacteroidetes; c__Bacteroidia; o__Bacteroidales; f__Rikenellaceae; g__unclassified_Rikenellaceae; s__unclassified_Rikenellaceae* |
| ASV_36434 | *d__Bacteria; p__Bacteroidetes; c__Bacteroidia; o__Bacteroidales; f__S24-7; g__unidentified_S24-7; s__unidentified_S24-7* |
| ASV_34421 | *d__Bacteria; p__Bacteroidetes; c__Bacteroidia; o__Bacteroidales; f__Rikenellaceae; g__Alistipes; s__unclassified_Alistipes* |
| ASV_16599 | *d__Bacteria; p__Bacteroidetes; c__Bacteroidia; o__Bacteroidales; f__S24-7; g__unidentified_S24-7; s__unidentified_S24-7* |
| ASV_6357 | *d__Bacteria; p__Bacteroidetes; c__Bacteroidia; o__Bacteroidales; f__Rikenellaceae; g__unclassified_Rikenellaceae; s__unclassified_Rikenellaceae* |
| ASV_45194 | *d__Bacteria; p__Proteobacteria; c__Gammaproteobacteria; o__Enterobacteriales; f__Enterobacteriaceae; g__unclassified_Enterobacteriaceae; s__unclassified_Enterobacteriaceae* |
| ASV_28188 | *d__Bacteria; p__Proteobacteria; c__Gammaproteobacteria; o__Enterobacteriales; f__Enterobacteriaceae; g__unclassified_Enterobacteriaceae; s__unclassified_Enterobacteriaceae* |
| ASV_18052 | *d__Bacteria; p__Bacteroidetes; c__Bacteroidia; o__Bacteroidales; f__Rikenellaceae; g__unidentified_Rikenellaceae; s__unidentified_Rikenellaceae* |
| ASV_63974 | *d__Bacteria; p__Firmicutes; c__Clostridia; o__Clostridiales; f__Lachnospiraceae; g__unclassified_Lachnospiraceae; s__unclassified_Lachnospiraceae* |
| ASV_32173 | *d__Bacteria; p__Firmicutes; c__Clostridia; o__Clostridiales; f__unidentified_Clostridiales; g__unidentified_Clostridiales; s__unidentified_Clostridiales* |
| ASV_17692 | *d__Bacteria; p__Firmicutes; c__Bacilli; o__Lactobacillales; f__Lactobacillaceae; g__Lactobacillus; s__unclassified_Lactobacillus* |
| ASV_55089 | *d__Bacteria; p__Proteobacteria; c__Betaproteobacteria; o__Burkholderiales; f__Comamonadaceae; g__unclassified_Comamonadaceae; s__unclassified_Comamonadaceae* |
| ASV_68279 | *d__Bacteria; p__Bacteroidetes; c__Bacteroidia; o__Bacteroidales; f__S24-7; g__unidentified_S24-7; s__unidentified_S24-7* |
| ASV_43310 | *d__Bacteria; p__Proteobacteria; c__Alphaproteobacteria; o__Rhodospirillales; f__Rhodospirillaceae; g__Novispirillum; s__unidentified_Novispirillum* |
| ASV_16680 | *d__Bacteria; p__Bacteroidetes; c__Bacteroidia; o__Bacteroidales; f__S24-7; g__unidentified_S24-7; s__unidentified_S24-7* |
| ASV_44250 | *d__Bacteria; p__Proteobacteria; c__Alphaproteobacteria; o__Sphingomonadales; f__Sphingomonadaceae; g__unclassified_Sphingomonadaceae; s__unclassified_Sphingomonadaceae* |
| ASV_22822 | *d__Bacteria; p__Proteobacteria; c__Alphaproteobacteria; o__Rhizobiales; f__unclassified_Rhizobiales; g__unclassified_Rhizobiales; s__unclassified_Rhizobiales* |
| ASV_6350 | *d__Bacteria; p__Verrucomicrobia; c__Verrucomicrobiae; o__Verrucomicrobiales; f__Verrucomicrobiaceae; g__Akkermansia; s__Akkermansia_muciniphila* |
| ASV_24822 | *d__Bacteria; p__Proteobacteria; c__Betaproteobacteria; o__Burkholderiales; f__Comamonadaceae; g__unclassified_Comamonadaceae; s__unclassified_Comamonadaceae* |
| ASV_71083 | *d__Bacteria; p__Firmicutes; c__Bacilli; o__Bacillales; f__unclassified_Bacillales; g__unclassified_Bacillales; s__unclassified_Bacillales* |
| ASV_53464 | *d__Bacteria; p__Proteobacteria; c__Gammaproteobacteria; o__Pseudomonadales; f__Moraxellaceae; g__Acinetobacter; s__Acinetobacter_guillouiae* |
| ASV_50035 | *d__Bacteria; p__Bacteroidetes; c__Bacteroidia; o__Bacteroidales; f__unclassified_Bacteroidales; g__unclassified_Bacteroidales; s__unclassified_Bacteroidales* |
| ASV_69694 | *d__Bacteria; p__Bacteroidetes; c__Bacteroidia; o__Bacteroidales; f__S24-7; g__unidentified_S24-7; s__unidentified_S24-7* |
| ASV_16010 | *d__Bacteria; p__[Thermi]; c__Deinococci; o__Thermales; f__Thermaceae; g__Thermus; s__unidentified_Thermus* |
| ASV_48409 | *d__Bacteria; p__Proteobacteria; c__Gammaproteobacteria; o__Pseudomonadales; f__Moraxellaceae; g__Acinetobacter; s__Acinetobacter_rhizosphaerae* |
| ASV_6748 | *d__Bacteria; p__Proteobacteria; c__Deltaproteobacteria; o__Desulfovibrionales; f__Desulfovibrionaceae; g__Desulfovibrio; s__Desulfovibrio_C21_c20* |
| ASV_24558 | *d__Bacteria; p__Proteobacteria; c__Gammaproteobacteria; o__Pseudomonadales; f__Moraxellaceae; g__Alkanindiges; s__Alkanindiges_illinoisensis* |
| ASV_55528 | *d__Bacteria; p__Bacteroidetes; c__Cytophagia; o__Cytophagales; f__Cytophagaceae; g__Spirosoma; s__unidentified_Spirosoma* |
| ASV_49245 | *d__Bacteria; p__Proteobacteria; c__Betaproteobacteria; o__Burkholderiales; f__Comamonadaceae; g__unclassified_Comamonadaceae; s__unclassified_Comamonadaceae* |
| ASV_53789 | *d__Bacteria; p__Proteobacteria; c__Gammaproteobacteria; o__Pseudomonadales; f__Moraxellaceae; g__Acinetobacter; s__Acinetobacter_johnsonii* |
| ASV_71126 | *d__Bacteria; p__Proteobacteria; c__Gammaproteobacteria; o__Pseudomonadales; f__Moraxellaceae; g__Alkanindiges; s__Alkanindiges_illinoisensis* |
| ASV_23238 | *d__Bacteria; p__Proteobacteria; c__Betaproteobacteria; o__Burkholderiales; f__Alcaligenaceae; g__Sutterella; s__unidentified_Sutterella* |
| ASV_6028 | *d__Bacteria; p__Bacteroidetes; c__Bacteroidia; o__Bacteroidales; f__unidentified_Bacteroidales; g__unidentified_Bacteroidales; s__unidentified_Bacteroidales* |
| ASV_61196 | *d__Bacteria; p__Firmicutes; c__Clostridia; o__Clostridiales; f__unidentified_Clostridiales; g__unidentified_Clostridiales; s__unidentified_Clostridiales* |
| ASV_3140 | *d__Bacteria; p__Proteobacteria; c__Gammaproteobacteria; o__Pseudomonadales; f__Moraxellaceae; g__Acinetobacter; s__Acinetobacter_rhizosphaerae* |
| ASV_1521 | *d__Bacteria; p__Firmicutes; c__Clostridia; o__Clostridiales; f__Veillonellaceae; g__Phascolarctobacterium; s__unidentified_Phascolarctobacterium* |
| ASV_22044 | *d__Bacteria; p__TM7; c__TM7-3; o__CW040; f__F16; g__unidentified_F16; s__unidentified_F16* |
| ASV_8146 | *d__Bacteria; p__Proteobacteria; c__Alphaproteobacteria; o__Rhodospirillales; f__Rhodospirillaceae; g__Elstera; s__Elstera_litoralis* |
| ASV_20735 | *d__Bacteria; p__Firmicutes; c__Clostridia; o__Clostridiales; f__Clostridiaceae; g__Candidatus_Arthromitus; s__unidentified_Candidatus_Arthromitus* |
| ASV_58426 | *d__Bacteria; p__Firmicutes; c__Bacilli; o__Lactobacillales; f__Lactobacillaceae; g__Lactobacillus; s__unidentified_Lactobacillus* |


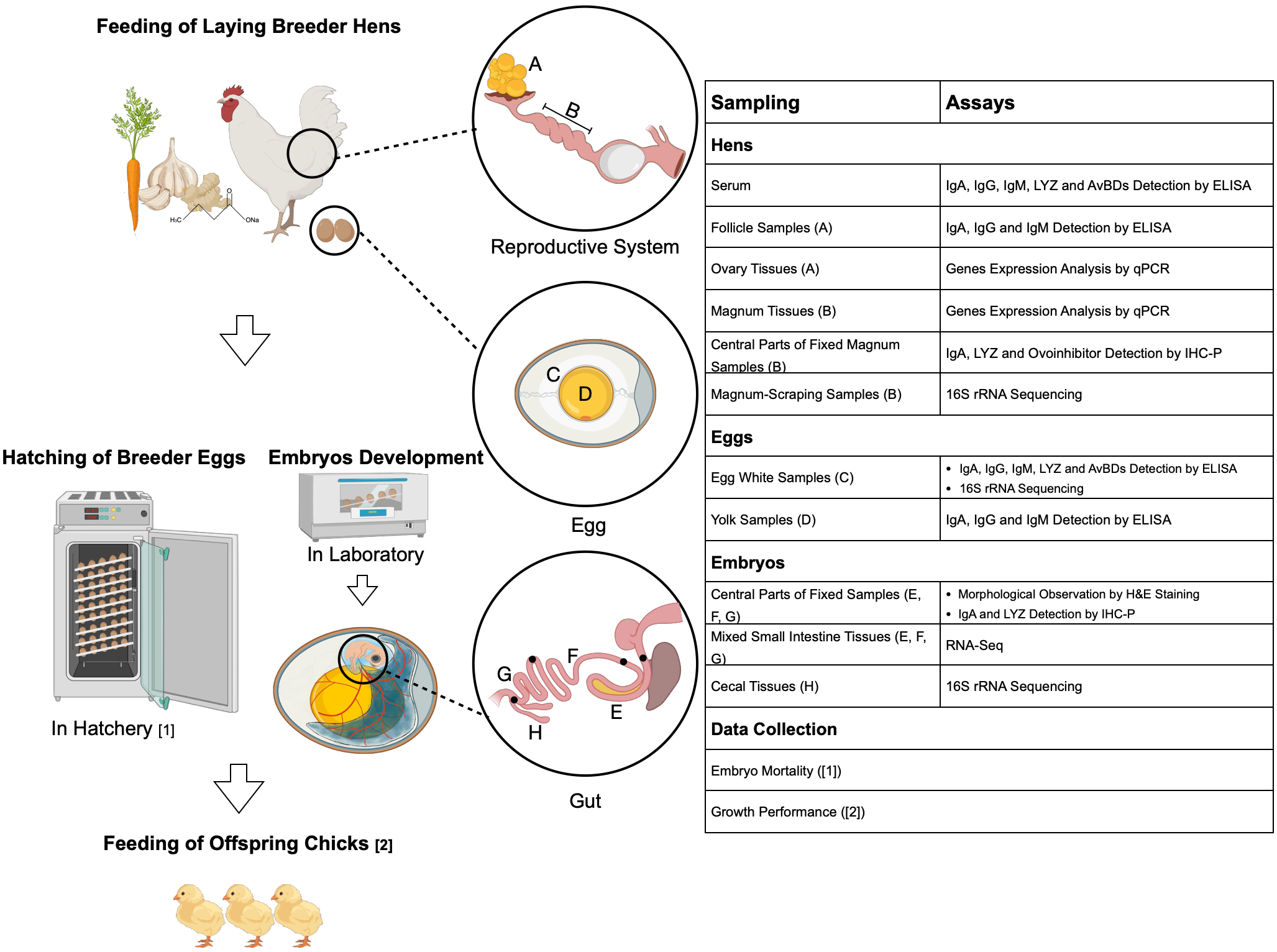


**Figure S1.** Experimental design.


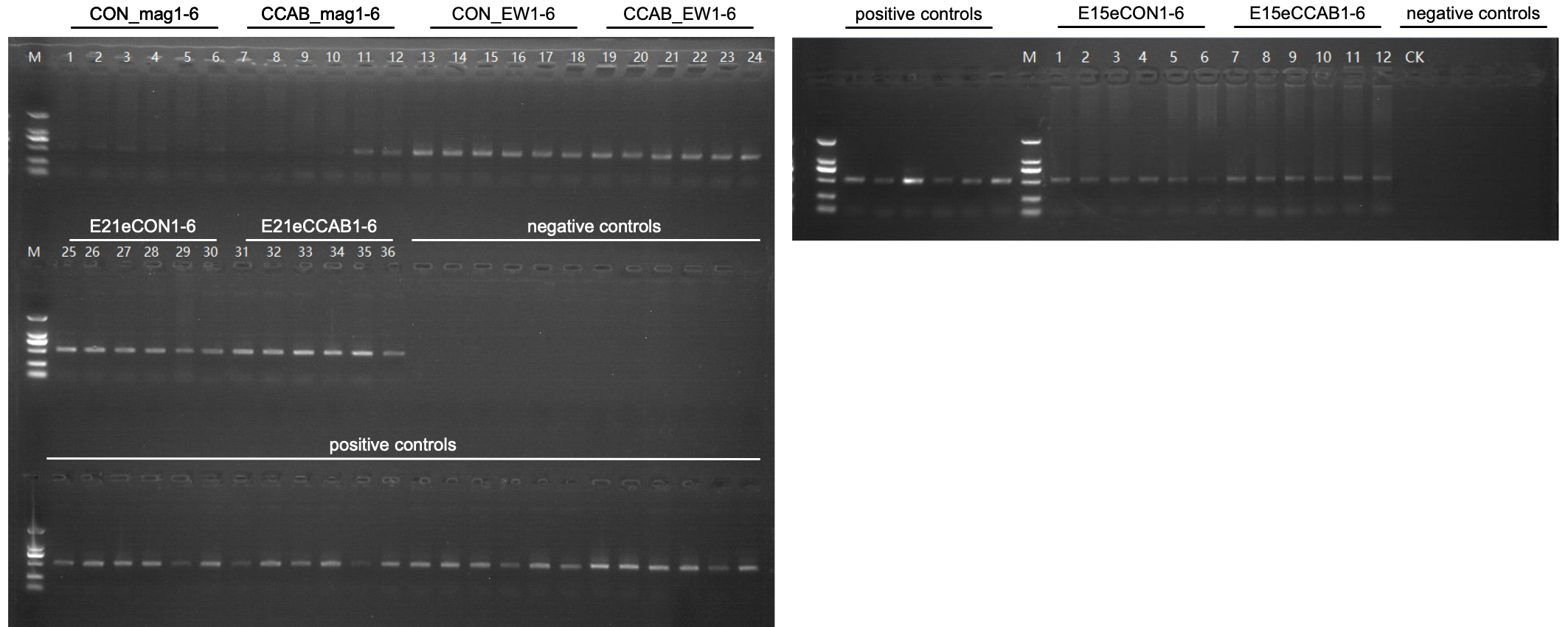


**Figure S2.** Electrophoresis results of PCR amplification products with positive and negative controls. Marker = 2000, 1000, 750, 500, 250 and 100 bp from top to bottom (TaKaRa, DL2000 DNA Marker).


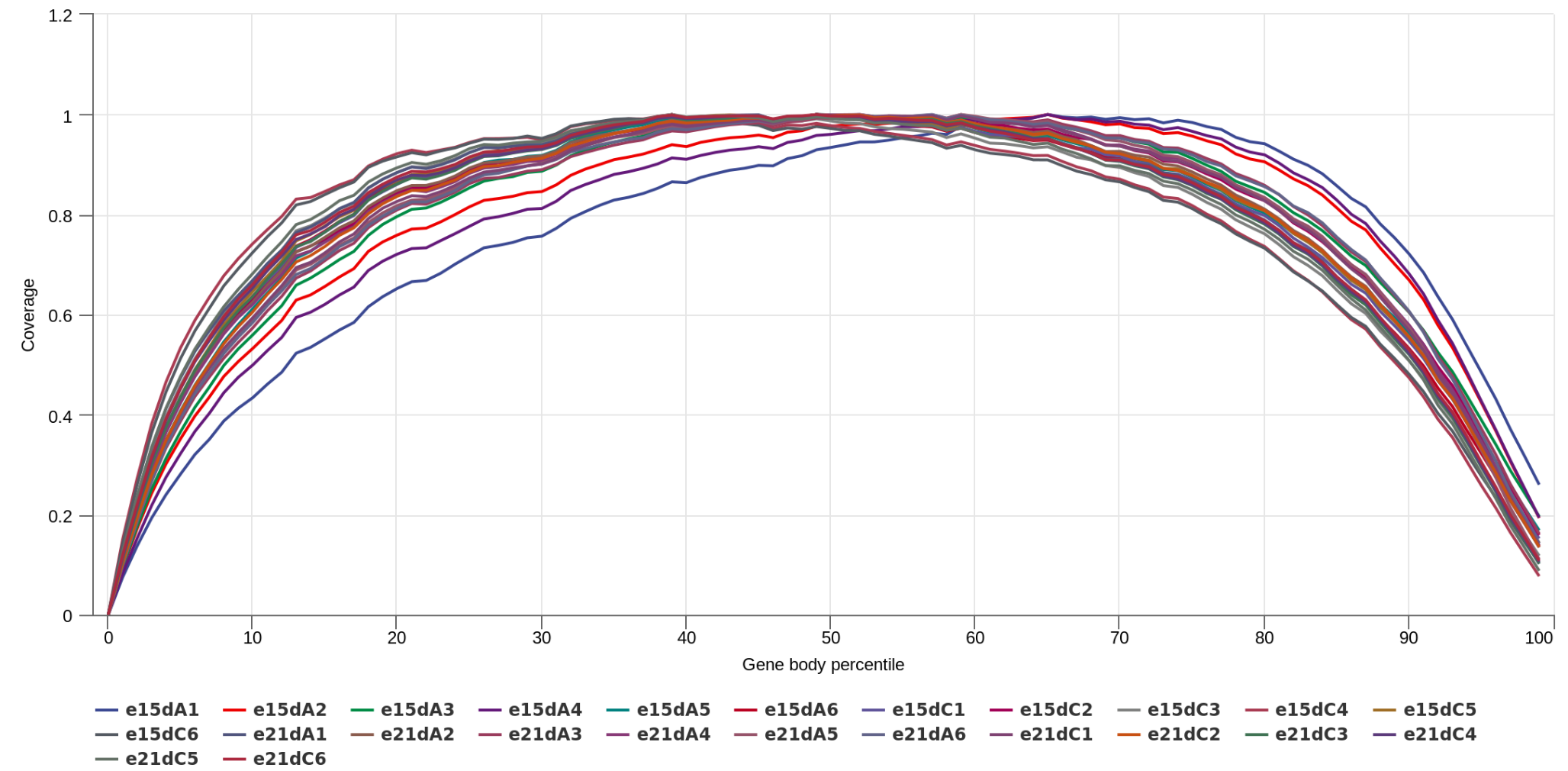


**Figure S3.** Gene coverage of each sample for mapping quality control. CON and CCAB are abbreviated as A and C in the figure.


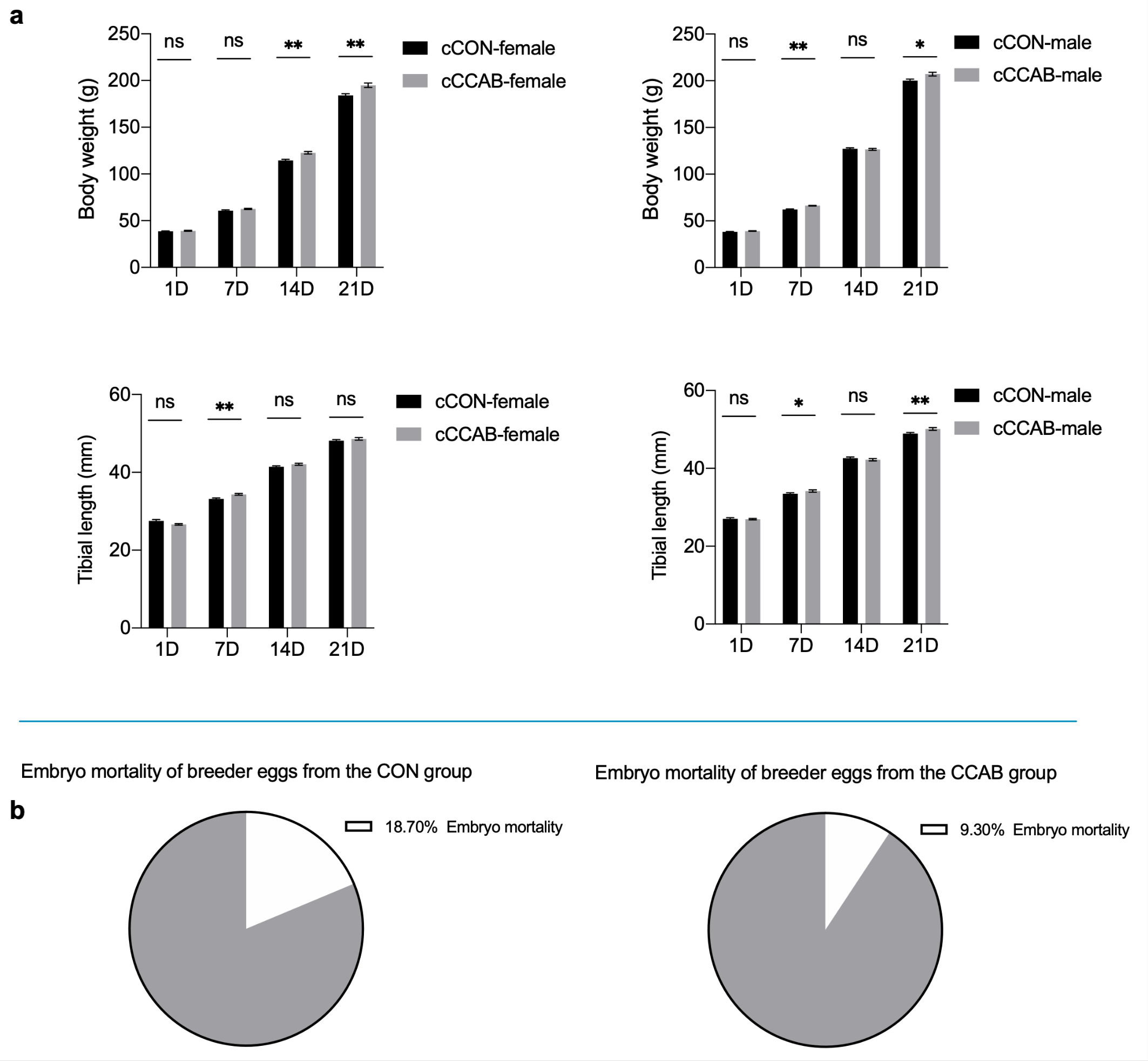


**Figure S4.** (**a**). Growth performance of the offspring chicks from 1 to 21 d of age. Data are shown as the means + SEMs. Student’s t test was conducted. ns *P* ≥ 0.05, **P <* 0.05 and ***P <* 0.01. n = 30 chicks. (**b**) Embryo mortality in the CON (left) and CCAB (right) groups. Embryo mortality = dead embryo number/fertile egg number. Set egg number = 900 eggs in each group. The number of fertile eggs was 792 and 797 in the CON and CCAB groups, respectively.


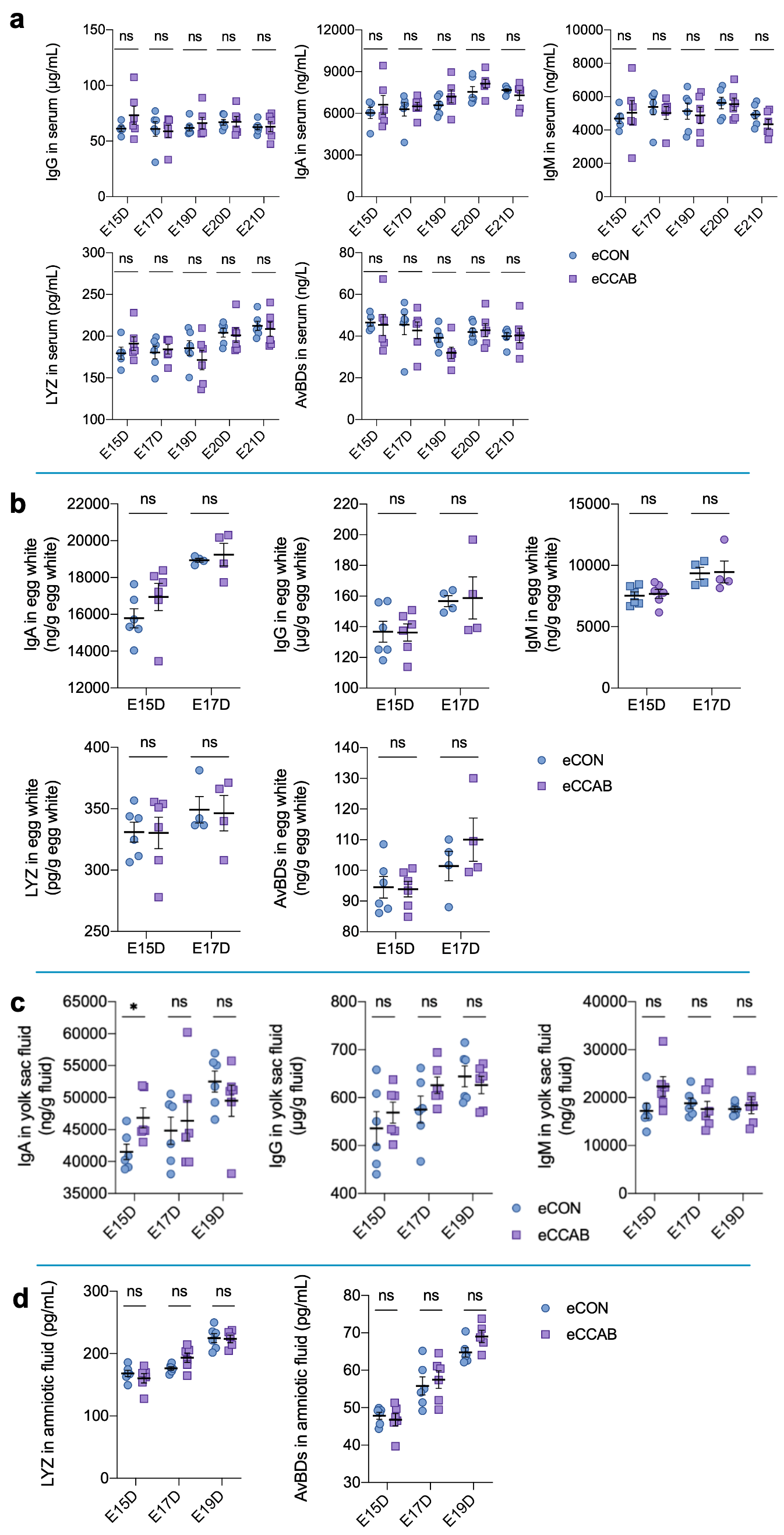


**Figure S5.** IgA, IgG, IgM, LYZ and AvBDs levels in embryonic serum (**a**) and egg white (**b**); the levels of IgA, IgG and IgM in embryonic yolk sac fluid (**c**); and the levels of LYZ and AvBDs in amniotic fluid (**d**) as determined by ELISA. Data are means ± SEMs. Student’s t test was conducted. ns *P* ≥ 0.05 and **P <* 0.05.


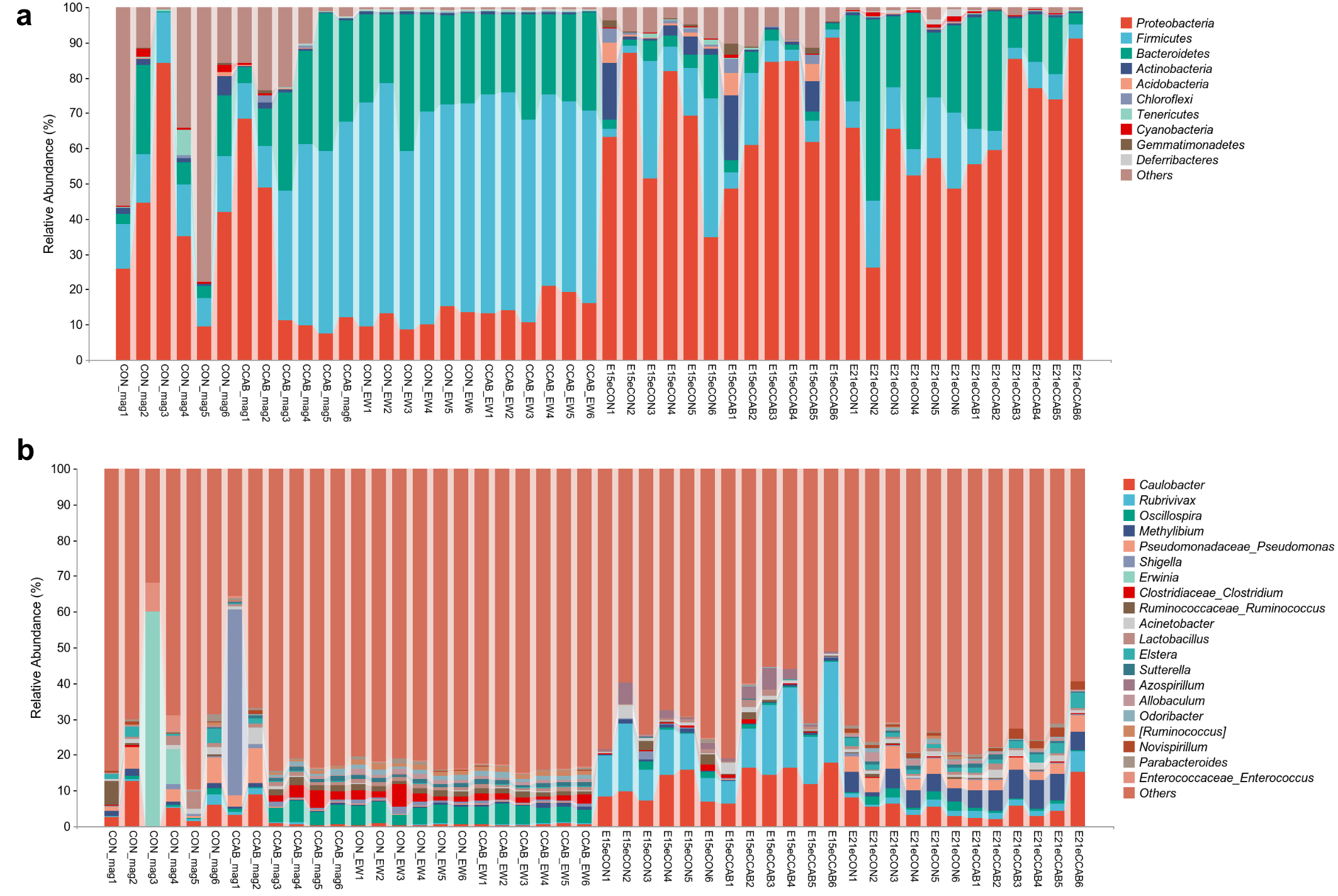


**Figure S6.** Microbial composition at the phylum (**a**) and genus (**b**) levels in the magnum (abbreviated as mag), egg white (abbreviated as EW), and E15 and E21 embryonic gut in each sample.


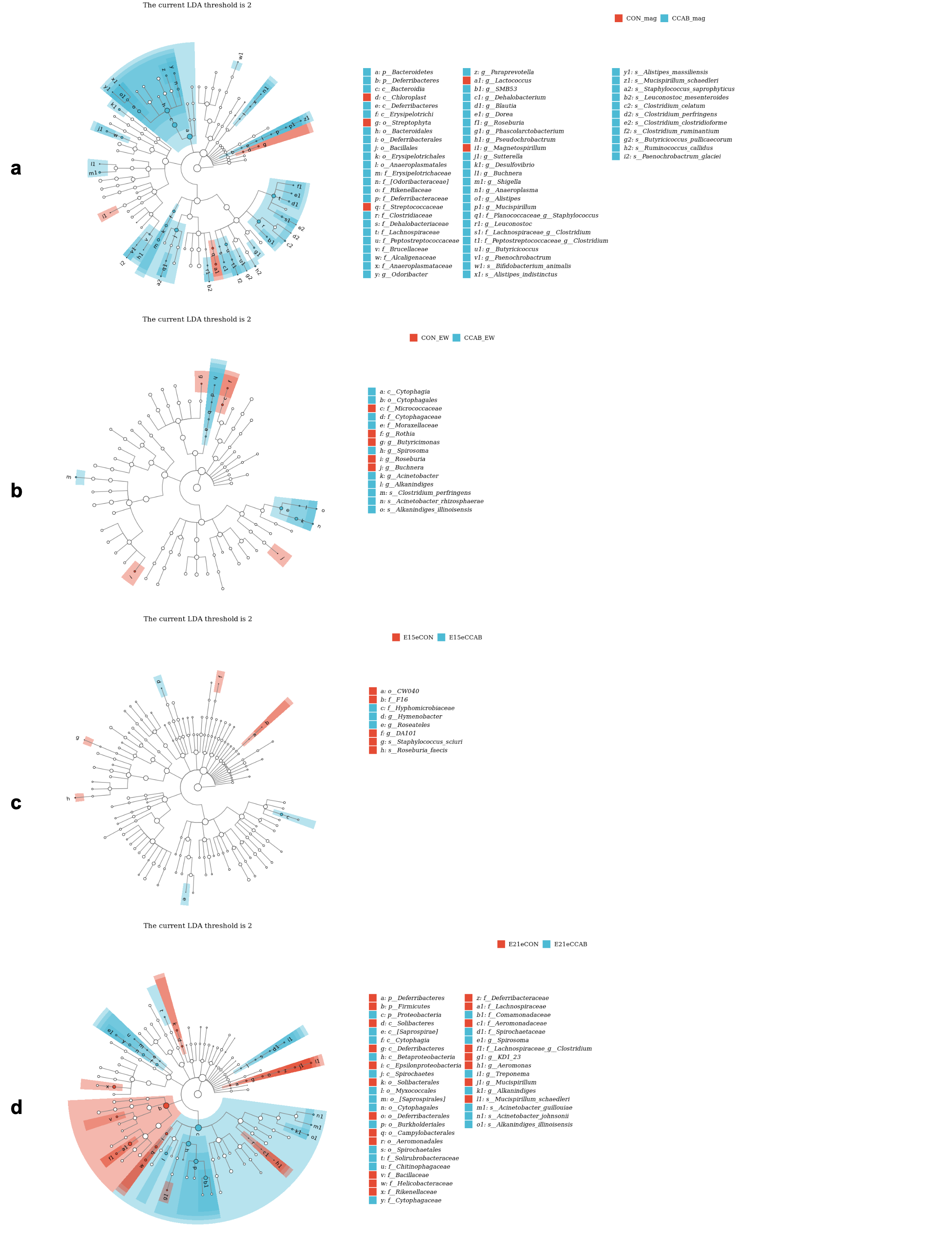


**Figure S7.** Significantly enriched taxa (log_10_(LDA score) > 2, *P* < 0.05) in the magnum (**a**; abbreviated as mag), egg white (**b**; abbreviated as EW), and embryonic gut at E15 (**c**) and E21 (**d**) between groups as determined by LEfSe with Kruskal‒Wallis and Wilcoxon tests and the one-against-all strategy.
